# Global dengue importation: a systematic review

**DOI:** 10.1186/s12879-021-06740-1

**Published:** 2021-10-19

**Authors:** Xiao Wei Sylvia Gwee, Pearleen Ee Yong Chua, Junxiong Pang

**Affiliations:** 1 Saw Swee Hock School of Public Health, National University of Singapore, National University Health System, Singapore, Singapore; 2 Centre for Infectious Disease Epidemiology and Research, National University of Singapore, Singapore, Singapore; 3 Tahir Foundation Building, National University Singapore, 12 Science Drive 2, #10-01, Singapore, 117549 Singapore

**Keywords:** Global health, Dengue, Importation, Outbreak, Public health

## Abstract

**Background:**

Importation of dengue following globalization presents an emerging threat to global health. However, evidence on global geographical sources and the potential of dengue importation globally are lacking. This study aims to systematically review the sources of dengue importation globally and the risk of dengue outbreaks globally.

**Methods:**

This systematic review was conducted in accordance to Cochrane’s PRISMA guidelines. Articles published through 31 December 2019 with laboratory-confirmed dengue imported cases were consolidated from PubMed, EMBASE and Scopus. Sources of dengue importation reported worldwide were analysed by country and geographical regions. Sources of dengue importation into United States of America and Europe specifically were also analysed.

**Results:**

A total of 3762 articles were found. Among which, 210 articles—documenting 14,972 imported dengue cases with reported sources—were eligible. 76.3% of imported cases worldwide were from Asia. 15.7%, 5.6%, 2.0% and 0.1% were imported from the Americas, Africa, Oceania and Europe regions respectively. Imported dengue cases in the U.S. were from Americas (55.3%), Asia (34.7%), Africa (6.7%) and Oceania (3.3%). Imported dengue cases in Europe were from Asia (66.0%), Americas (21.9%), Africa (10.8%) and Oceania (1.1%).

**Conclusion:**

The potential of dengue outbreaks occurring globally, especially among non-endemic regions with dengue-susceptible populations is high. With the expansion of Aedes mosquito population globally due to global warming and globalisation, dengue importation constitutes an emerging global health security threat.

**Supplementary Information:**

The online version contains supplementary material available at 10.1186/s12879-021-06740-1.

## Background

Dengue fever is a mosquito-borne infectious disease that is prevalent in the tropical and sub-tropical regions. Today, approximately 3.6 billion people live in areas at risk of transmission. Dengue virus (DENV) has been estimated to cause up to 390 million infections and 96 million symptomatic cases annually [1]. A virus of the flaviridae family, dengue virus has four serologically distinct phenotypes, DENV1/2/3/4 [2]. Transmission occurs mainly via *Aedes aegypti* and *Aedes albopictus*, which are also highly prevalent in the tropical and sub-tropical regions [2].

### Dengue fever

Dengue fever is characterized by an incubation period ranging from 3 to 14 days, lasting 5–7 days on average [3]. The viremic human host is capable of transmitting the virus via the mosquito vector for 5–12 days after infection. DENV infections are largely asymptomatic, with only 20% of infections presenting febrile illness accompanied by general symptoms like joint and muscle pain, skin rashes, nausea, severe headache [3]. While the classic dengue fever is usually self-limiting, a minority can be threatened by severe complications like dengue haemorrhagic fever or dengue shock syndrome, possibly leading to fatal outcomes [4]. Dengue is a mandatory notifiable disease in many countries under the recommendation of the International Health Regulation [5]. However, majority of infected cases, being asymptomatic, are not tracked in the global surveillance system despite possessing the potential of transmitting dengue virus [6].

### International travel

As of this decade, more than 125 countries globally are dengue endemic, with transmission documented in every World Health Organisation (WHO) region [7]. However, cases can be reported in endemic and non-endemic countries alike due to importation of viremic travellers made possible by international travel [8, 9]. Travellers to the tropical region can acquire the infection through bites by the *Aedes aegypti*, which is largely responsible for the spread of dengue fever in tropical regions [10], while transmission in non-tropical regions occurs mainly via *Aedes albopictus*, a secondary vector with a larger range of biting targets including birds, cats, dogs and other mammals [10–12]. The speed of travelling via planes and trains has enabled the transfer of viremic travellers from a disease endemic region to a non-endemic region. Coupled with an increasing presence of competent vectors in non-endemic regions due to global warming [1, 13] and globalisation [14], viremic travellers can act as potential virus host source for transmission to local susceptible populations. Yet, diagnosis of dengue is often impeded by its mild and undifferentiated symptoms [15], delaying treatment and actions against dengue transmission. This marks the origin of an imported index case making for autochthonous case transmission that could potentially spiral into an outbreak [4]. Endemicity is subsequently facilitated by conducive breeding sites in crowded urban communities, lack of vector control, climate change and vector adaptation [4, 10]

Due to the emerging global impact of imported dengue, a good understanding of the trend of imported dengue in terms of its potential geographical sources will be highly relevant to inform policy, risk assessment and intervention-decision making process to prevent and to delay dengue outbreaks. Therefore, this systematic review aims to give a descriptive analysis of the sources of global importation of dengue by establishing a baseline summary of cases imported by travellers across borders. In addition, this review focuses on dengue importation into the United States and Europe, due to increasing threat of local dengue outbreaks in these dengue non-endemic regions in the last decade.

## Methods

### Search strategy

A systematic review was performed according to the Preferring Reporting Items for Systematic Reviews and Meta-Analyses (PRIMSA) guidelines. Key search terms “travellers, “travel”, “imported” and “dengue” were used to systematically search PubMed, Scopus, EMBASE and Cochrane for English and Chinese language articles related to cross-border importation of dengue worldwide with no restriction on countries. The two languages were chosen as all authors are proficient in English and Chinese. Articles published through 31 December 2019 were included. Search strings and returns are appended in full in the appendix. GXWS applied the inclusion criteria to all studies identified through the database searches. CEYP and JP independently did the eligibility assessment using the inclusion criteria for all of the studies at the initial screening of titles, abstracts, and full texts. Assessment of agreement was undertaken at each of these stages, with any disagreement resolved by consensus after referring to the protocol. Ethics approval is not required as only aggregate data was used from the selected published articles. In addition, there is no personal or personal medical data involved in this systematic review.

### Selection criteria

Examined publications include surveillance reports, case-series, case reports and phylogenetic studies. All articles with laboratory-confirmed cases of dengue imported by travellers were included. Laboratory confirmed cases entail those defined by the original authors of the accepted publications and also probable cases (mostly positive by serology) that were grouped together as confirmed cases in the publications. Source and time period of articles from each country were then manually aligned to remove potential overlapping cases.

The key inclusion criteria was an imported case of dengue—an individual diagnosed with dengue following recent travel history. However, definitions of confirmed and probable cases lacked uniformity across studies in that cases diagnosed using the same methods were found to be defined differently at times. In order to present only true dengue cases, this review only included laboratory confirmed cases and probable cases which fit the United States (U.S.) CDC criteria for dengue confirmation [16]—cases confirmed through RT-PCR, virus isolation and serological means. The following were excluded: Cases mentioned as reported or diagnosed without laboratory confirmation, publications that do not report specific numbers of imported dengue cases, interstate importation of dengue, and cases associated with unclear travel history. Details on the inclusion process and PRISMA flowchart can be found in the Additional file 1.

### Data extraction and analysis

Information on cases’ origin of travel and destination were extracted from all included publications. Geographical origin of travel and destination from data extracted were subsequently categorized into Africa, Americas, Asia, Caribbean, Europe and Oceania according to the United Nation (U.N.) geoscheme. In view of the diverse presentation of cases randomly reported across the time period, cases were analysed in whole regardless of reporting year. U.S. and Europe specific data were also extracted from the main dataset for a separate analysis. Chronological analysis was only performed for the U.S. specific data, which contain cases from 1977 to 2017.

Data analysis was performed using Microsoft Excel 2016. Descriptive statistics and graphical presentations were generated using Microsoft Excel 2016, while Tableau and Excel PowerMaps were employed in the making of geographical maps showcasing regional sources of dengue importation globally, into the U.S. and Europe, respectively, and the comparison of U.S. states reporting imported cases.

## Results

**Fig. 1:**
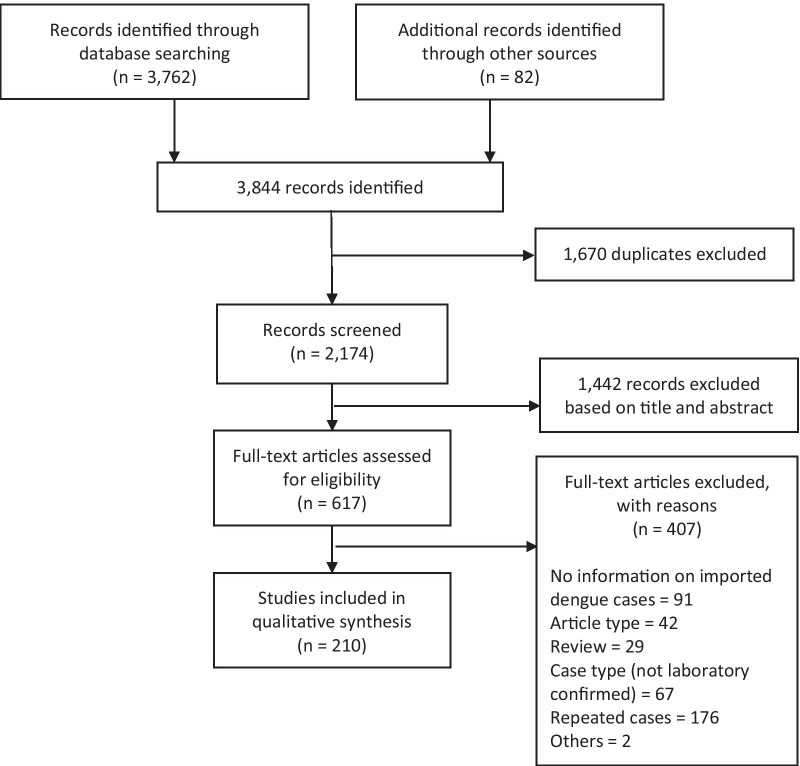
Flow of information through the search and screening phases of the review

The search returned a total of 3762 articles, with 2174 articles remaining after duplicates removal. Primary screening of the title and abstract of articles from database search and additional sources identified 617 relevant articles for full text screening. This systematic review eventually utilized data from 210 articles (Fig. 1).

### Global sources of dengue importation

**Fig. 2:**
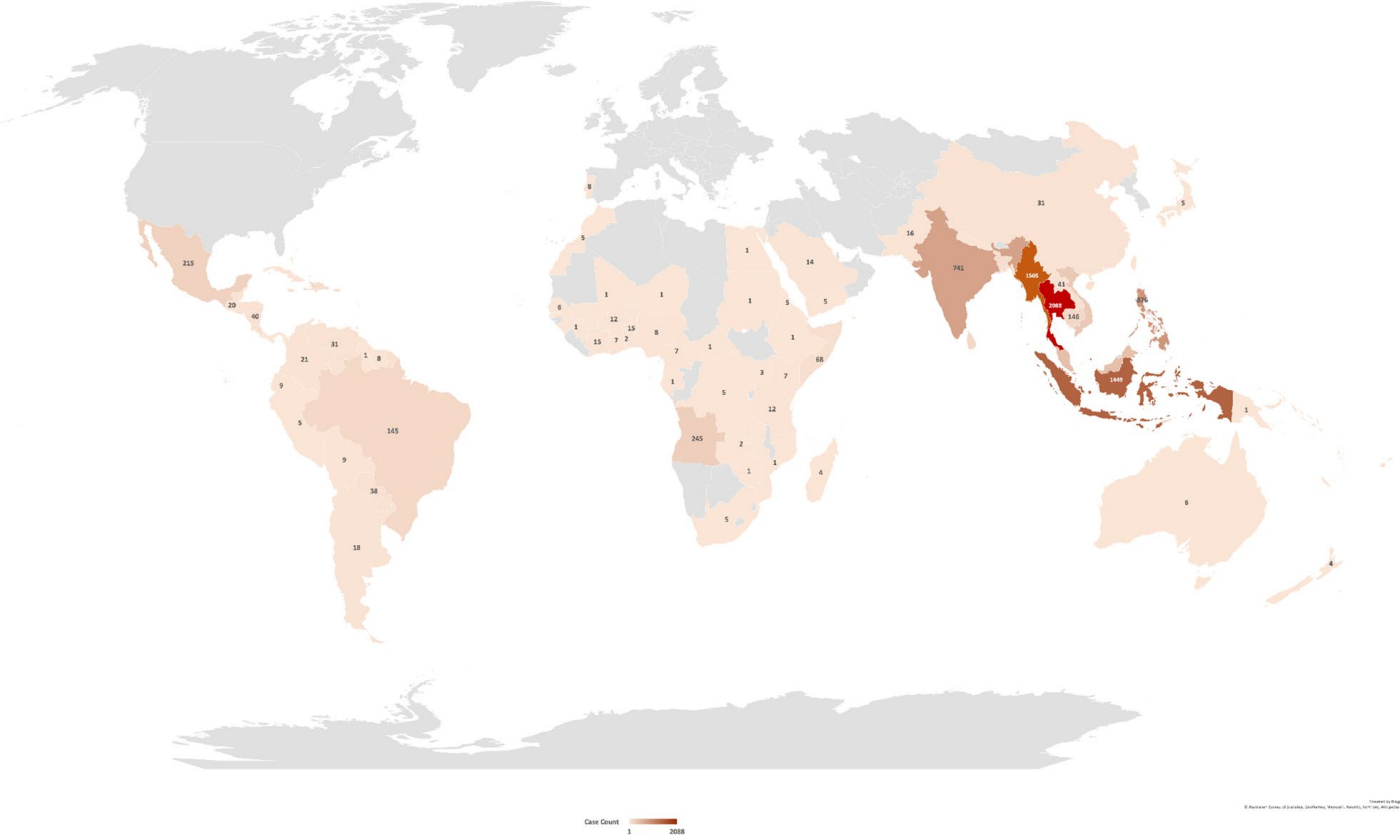
Reported global sources of imported dengue (created using Microsoft Excel Powermaps 2016)

Existing literature reported 30,405 cases of imported dengue fitting our inclusion criteria between 1951 and 2019. Specific origin of importation was unknown for 15,410 (50.7%) cases while 23 (0.08%) others were associated with travel to multiple regions. These two categories of cases, together with unspecified countries in each region were excluded from the mapping of sources from which dengue had been imported globally (Fig. 2). Of 14,972 cases with known origin, majority was attributed to Asia with 11,421 (76.3%) cases, while 2357 (15.7%) and 833 (5.6%) cases were imported from the Americas and Africa regions respectively (Fig. 2). Oceania and Europe only accounted for small proportions of cases at 297 (2.0%) and 14 (0.1%) each (Fig. 2).

Thailand, Myanmar, Indonesia and Philippines were the top Asian countries from which dengue cases were imported worldwide. Travel to these countries were associated with 2088 (18.3%), 1505 (13.2%), 1449 (12.7%) and 876 (7.7%) cases respectively (Fig. 2). For the Americas, Mexico is the principal source with 215 (9.1%) cases reported, followed by the Caribbean region and Brazil, with 211 (9.0%) and 145 (6.2%) cases of dengue associated respectively. Angola and Somalia were the most significant sources of imported dengue in the African region, accounting for 245 (27.7%) and 68 (7.7%) cases respectively, followed by Benin and Ivory Coast with 15 (1.7%) cases each. A detailed breakdown of the importation origins for cases worldwide is appended as Additional file 1: Table S1.

### Imported dengue in United States

**Fig. 3:**
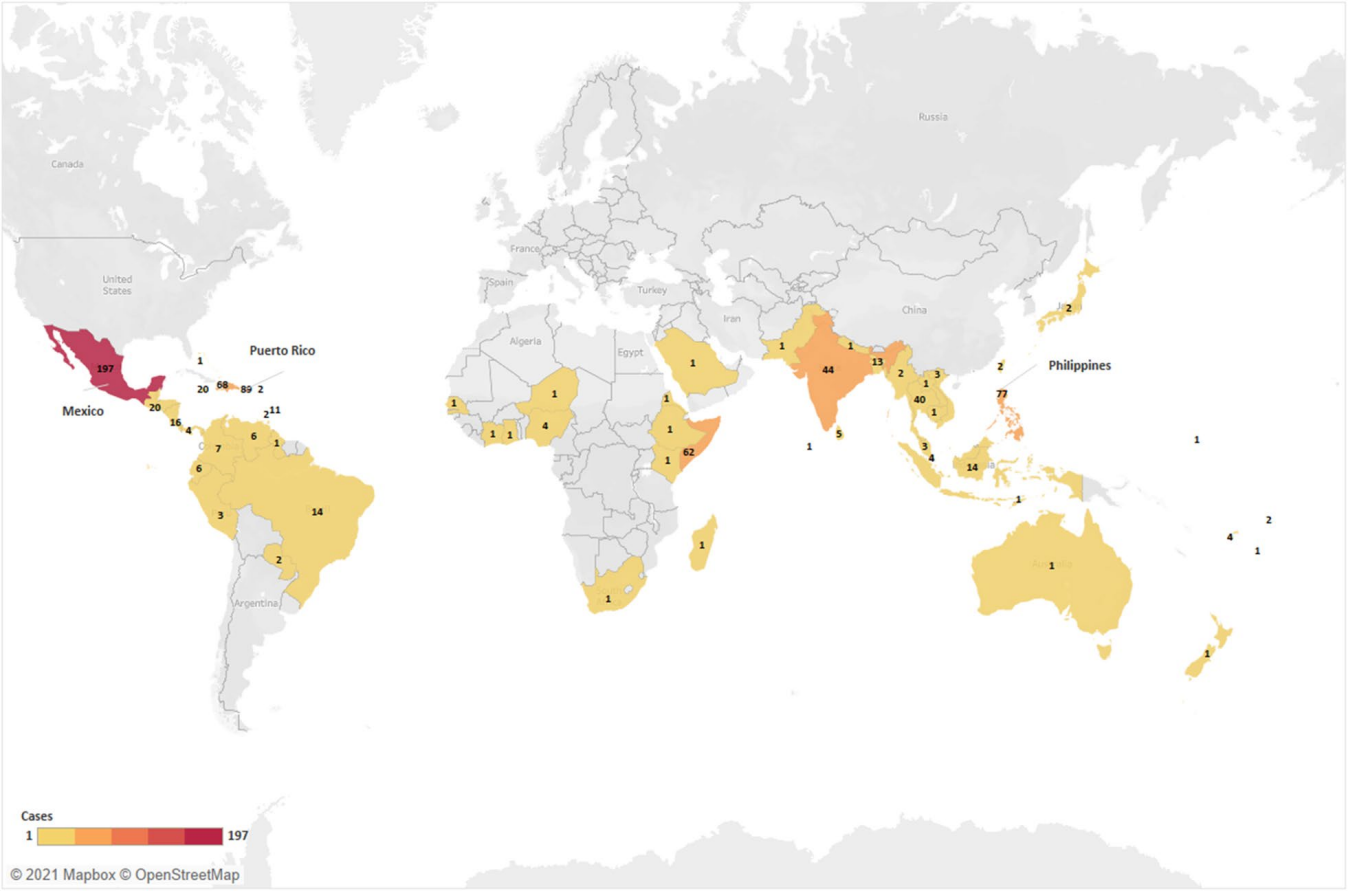
Geographical sources of imported dengue into US (created using Tableau 2021.1)

A total of 2476 cases were reported from 48 states of U.S. and the District of Colombia over the years 1977–2017. Majority of cases with known sources were imported from the Americas, totalling 846 (55.3%) cases. Dengue was associated with travel to Asia for 531 (34.7%) cases, Africa for 102 (6.7%) cases and Oceania for 50 (3.3%) cases (Fig. 3). Mexico and Puerto Rico were the top sources in Americas with 197 (23.3%) and 89 (10.5%) cases each (Fig. 3; Additional file 1: Table S2). In Asia, 77 (14.5%), 44 (8.3%) and 40 (7.5%) cases were associated with Philippines, India and Thailand respectively. African countries were more evenly distributed as import origins with the exception of Somalia.

**Fig. 4:**
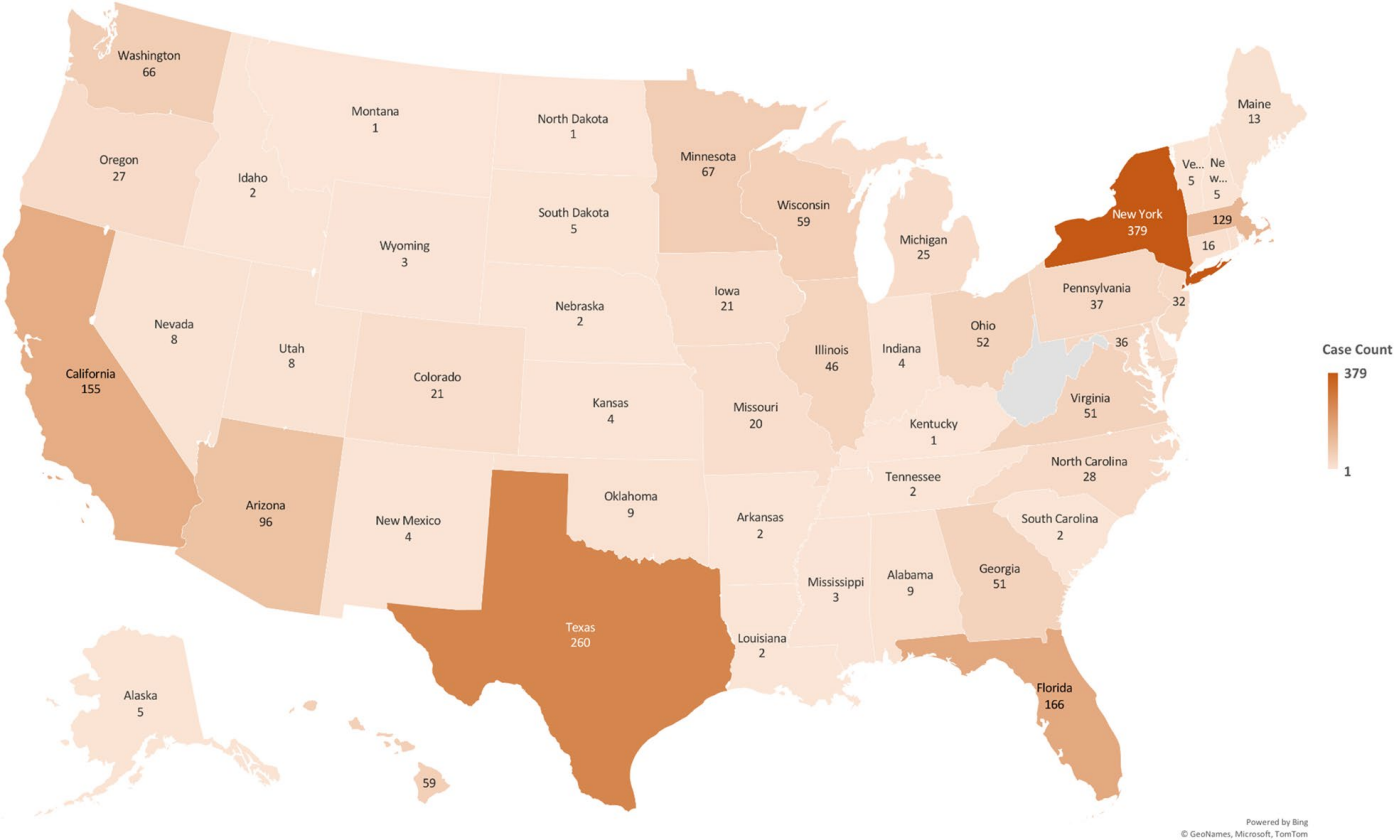
U.S. States reporting imported cases of dengue (1977–2017) (created using Microsoft Excel Powermaps 2016)

Of known reporting sources, New York tops the list of dengue reporting states with 379 cases (18.6%), followed by Texas, Florida, California, Massachusetts with 260 (12.8%), 166 (8.2%), 155 (7.6%) and 129 (6.3%) cases reported respectively (Fig. 4; Additional file 1: Table S3). Cases reported from New York were mainly from Dominican Republic (26; 20%), Puerto Rico (23; 17.7%) and Nicaragua (11; 8.5%) while Texas cases were mainly associated with Mexico (105; 62.9%) (Additional file 1: Table S4 and S5).

Cases were further stratified in decades to investigate potential temporal trends in dengue importation to the U.S. This analysis is only possible for the U.S. as their surveillance for dengue importation was documented consistently over the years. In general, dengue cases imported to the U.S. exhibited a steep increasing trend from 1970 to 2000s (Additional file 1: Table S6). Trend increased from 139 cases in 1977–1980s to 296 in the 1980s, 524 in the 1990s, eventually peaking at 960 in the 2000s before declining to 557 cases in 2010–2017. The increased trend of imported dengue is observed from both the Americas and Asia in the 1970–1990s (Additional file 1: Table S6 and S7).

### Imported dengue in Europe

**Fig. 5:**
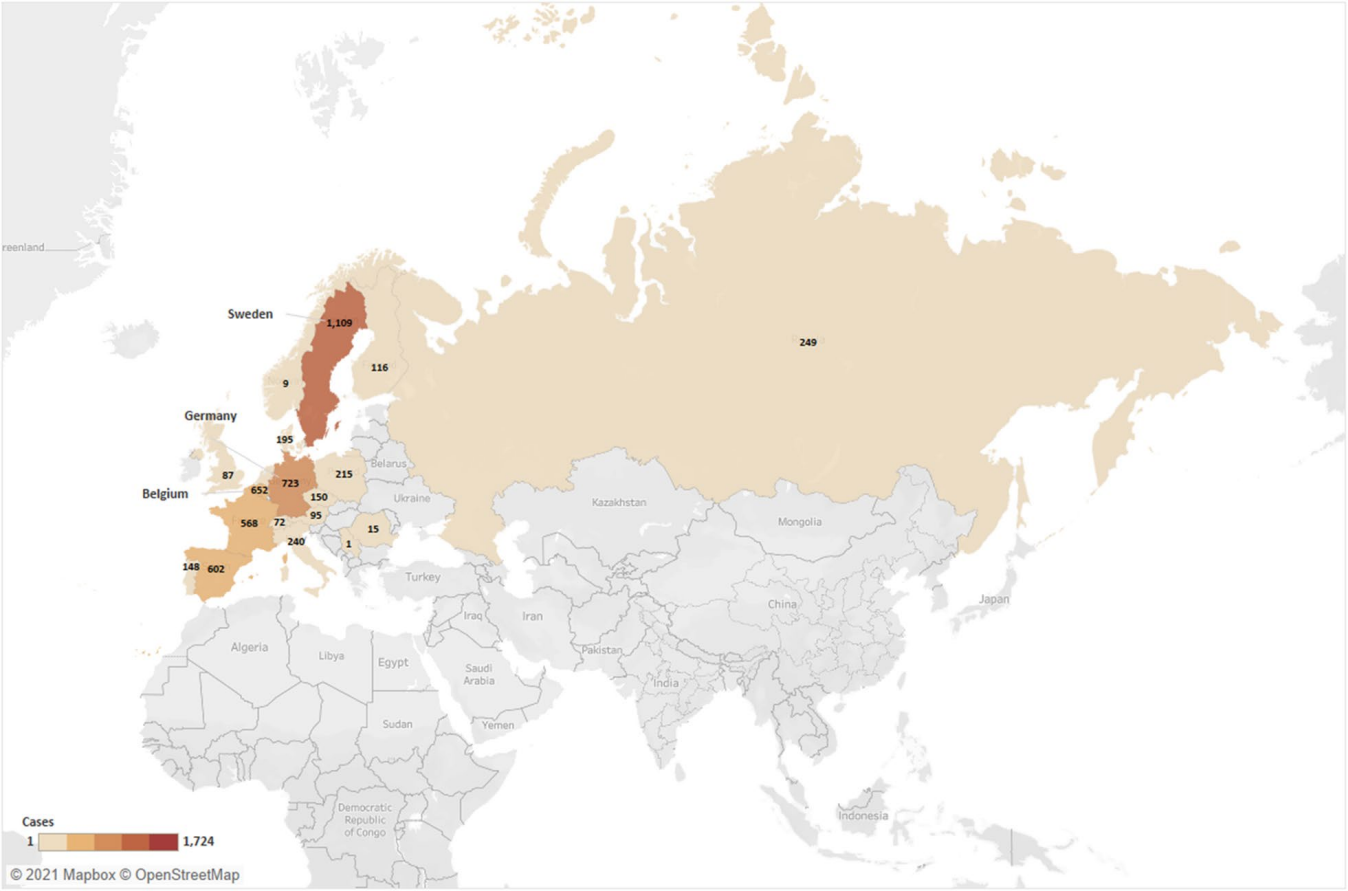
European countries reporting imported cases of dengue (created using Tableau 2021.1)

**Fig. 6:**
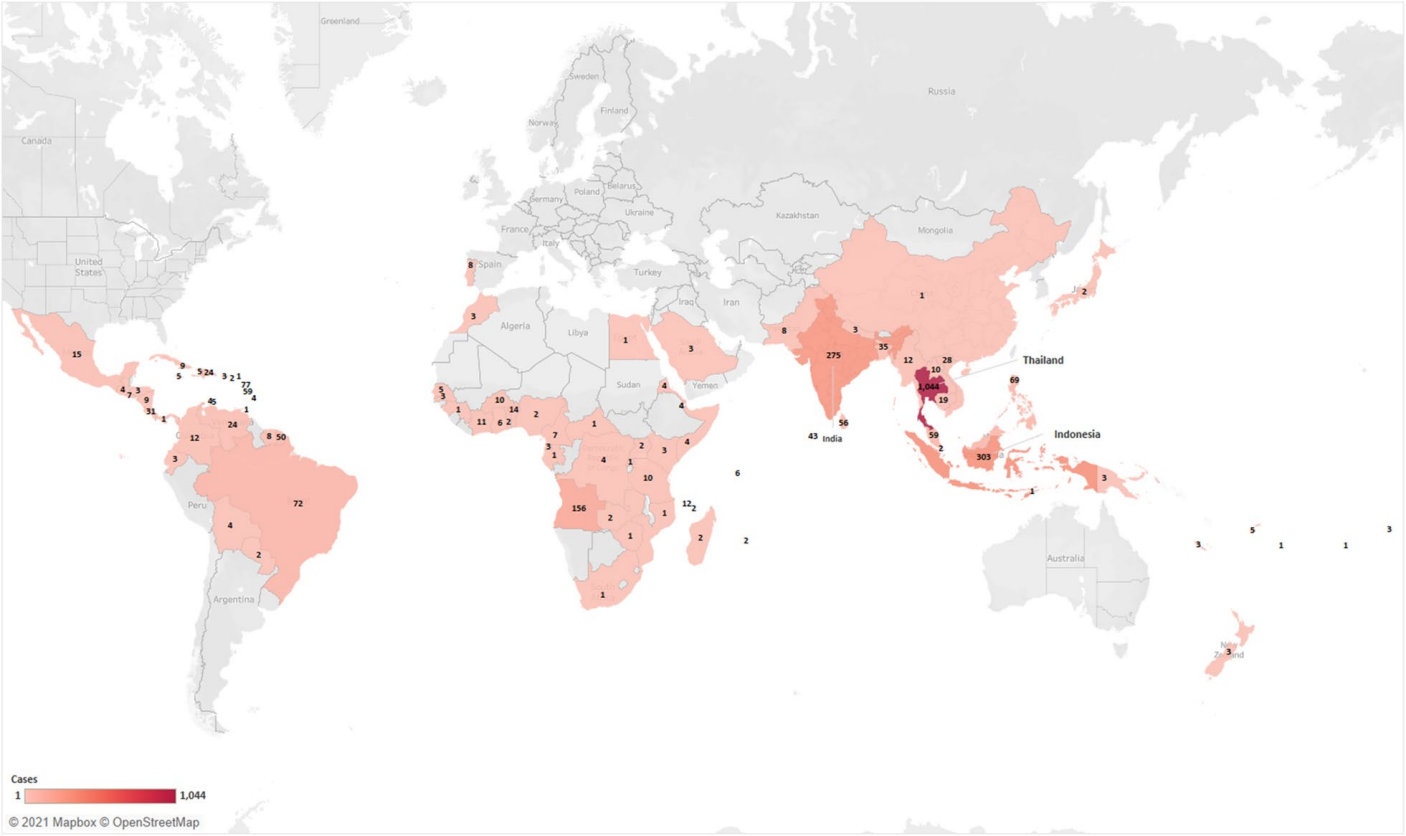
Sources of imported dengue into Europe (created using Tableau 2021.1)

A total of 7070 imported dengue cases were reported by European countries over the years. Amongst cases with known reporting sources, Sweden, Germany and Belgium recorded the highest trend of dengue importation with 1109 (20.7%), 723 (13.5%) and 652 (12.2%) cases respectively (Fig. 5; Additional file 1: Table S8). Of 4,177 cases with reported origin of import, there were 2758 (66.0%) from Asia, 915 (21.9%) from Americas, 452 (10.8%) from Africa, and 44 (1.1%) from Oceania (Fig. 6; Additional file 1: Table S9). Notably, Thailand was the greatest attributing source in Asia with 1044 cases, followed by Indonesia and India with 303 and 275 cases, respectively. There were also 8 cases imported from Portugal.

## Discussion

Our findings suggest Asia and the Americas as main regions from which travellers worldwide acquire dengue illness. Notably, Asia is found to be the main source of dengue importation into Europe while Latin America is the main source of dengue imported into the U.S. This observation could likely correspond to preferential traveling patterns of European travellers [17] and U.S. travellers to Asia.

Thailand, Indonesia and Philippines—where dengue has been a longstanding public health concern [18, 19]—are major sources of imported dengue in Asia. Epidemics were first recorded in the 1950s [18]. Rapid yet unplanned urbanization in these developing nations spawned inadequate water, sewage and waste management systems that produced favourable breeding habitats for the mosquito vectors [10, 18]. Sustained dengue transmission by highly domesticated mosquitoes since resulted in endemicity of dengue in these countries. In recent decades, they have evolved to become major tourist destinations. As of 2017, Southeast Asia saw the highest tourism growth among all regions, with Thailand ranked tenth globally in terms of both international tourist arrivals and tourism receipts [20]. Such places with high density of *Aedes* mosquitoes and/or high human population density increase the risk of dengue transmission [21, 22]. Hence, global travel not only amplifies the risk of dengue exposure for travellers to these regions, but also facilitate the speedy transport of these dengue carriers across borders as they return to their home countries infected [20, 23]. Notably, Myanmar is a major source of dengue importation only to China, as the border of China meets Myanmar at Yunnan. Most of the cases attributable to Myanmar were solely reported by China over a period of 12 years [24], testament to dengue importation’s association with preferential travelling patterns and its inadequacy as a standalone gauge of dengue transmission potential in a region.

Based on U.S. specific data, most of their imported cases originated from Mexico. This likely correlates with tourism preference, evident from the U.S. outbound tourism of almost 14 million travels to the Americas and Caribbean regions [25]. This preference is spurred by geographical proximity and allowance of visa free-entry for U.S. travellers to most Latin American countries for up to 90 days [17, 26]. In 2018, close to 37 million travels were made from U.S. to Mexico [25], while Mexico also ranked 2nd in terms of tourist arrivals to the U.S. with 18.5 million visitors [27]. Both outbound and inbound tourism trends point to Mexico as the country with which U.S. has the highest traffic of exchange, commensurate with the proportion of dengue cases associated with travel to Mexico. Most of the imported dengue cases in the U.S. were reported in New York, followed by Texas and Florida. All three states were highly popular with South American tourists, suggesting strong links between tourism and risk of dengue importation [28–33].

The peril of this observation lies in the widespread distribution of both mosquito vectors, *Aedes albopictus* and *Aedes aegypti* in Florida, while Texas, bordering Mexico which has long observed dengue activity, is one of the few U.S. states that have reported autochthonous dengue outbreaks [29, 34]. Such prominent vector presence translates to heightened risk of autochthonous transmission in their immune-susceptible resident population following the return of a viremic traveller.

While it is unlikely for one to acquire dengue illness in Europe, attention must be paid to the emerging threat posed by dengue in the region. This is especially so given Europe’s immense tourism popularity among Asians as well as Europeans’ increasing preference to travel to Asia [4]. According to WHO, autochthonous transmission was recorded in France and Croatia for the first time in 2010 [35–39] due to the presence of *Aedes albopictus* populations. The imported dengue cases from Portugal suggests the establishment of *Aedes albopictus* with sustainable local dengue transmission reported since 2012 [40, 41]. While Europe is recognised to be at low risk of dengue transmission [42, 43], dengue endemicity in Europe conveyed by *Aedes* establishment and increased imported dengue cases is not impossible with global warming producing more conducive environments for *Aedes* to transmit dengue [1, 13].

In Africa, Angola and Somalia accounted for the main bulk of imported cases among other African countries. This could be attributed to two key events; an outbreak that occurred in Angola in 2013 which implicated many countries worldwide [44] and Operation Restore Hope led by the U.S. troops to civil war-stricken Somalia in 1992, during which many soldiers acquired dengue [45]. Likewise, the low incidence of imported cases does not imply negligible dengue threat in the continent. Rather, there exists preferential traveling patterns to the region among travellers [46]. Additionally, dengue is likely under-recognized and under-reported in Africa. While our findings suggest endemicity of dengue transmission in Africa with local transmission reported among 22 out of 34 countries, official data for dengue prevalence remains unavailable for the region [46]. Inadequacies in the region’s dengue surveillance efforts is apparent. Since malaria is endemic to most African nations and treated as the pre-dominant cause of febrile illness, cases of dengue are often mis-diagnosed and mis-treated as malaria despite patient’s lack of response to anti-malarial drugs [46, 47]. Inadequacy in laboratory capacity also compromises differential laboratory diagnosis of febrile illnesses such as dengue [10, 46].

As media attention and medical resources divert to the COVID-19 pandemic, it is crucial not to neglect the persistent, if not escalating threat of dengue. The near complete shutdown of air travel likely ameliorated importation of dengue in the past few months, but any reduction is temporary as countries set up green travel lanes to recover their economies, or as international travel regains post pandemic. Furthermore, behavioral changes elicited by the COVID-19 pandemic drives local dengue transmission. Social distancing measures confined unprecedented numbers of the global population in their residences. This presents a greater susceptible population for Aedes aegypti, which predominately breeds in domestic habitats, consequently increasing dengue incidence [48]. In dengue-endemic areas, the local population’s risk increases twofold with this dual circulation of viruses. The possibility of coinfection with dengue and SARS-CoV-2 has been demonstrated in multiple case reports [49–54], one of which was in a returning traveller in Mayotte [50]. In all occurrences of coinfection aforementioned, cases were diagnosed with the second infection on a separate call back. Such misdiagnoses not only delay targeted treatment, thereby aggravating prognosis, but also cause community transmission of either diseases.

Given that early symptoms of both infections are similar, diagnostic workflow in dengue-endemic or tropical settings should harness rapid, accurate diagnostics that account for both viruses [54]. Clinicians ought to revise their triaging protocols for both dengue and SARS-CoV-2 infections depending on prevalence in the region or epidemiological history. Further research integrating diagnosis of both diseases via molecular or serological techniques can be beneficial. Research should project beyond the COVID-19 pandemic, to future proof diagnostics enabling detection of multiple diseases with typical symptoms. Vector control remains a vital dengue prevention measure and should be aggressively employed in dengue-endemic countries to prevent the catastrophic emergence of double epidemics, especially in resource poor ones. Overall, authorities should not lose sight of dengue as a long-term health issue and continue to monitor transmission and hotspots in conjunction with the COVID-19 pandemic progression.

This study is not without its limitations. Firstly, there is a likelihood of publication bias where only selected studies were published and archived in these databases, which directly implicates the cases available for consolidation. This is reflected in the example of Myanmar, whereby the large number of cases was a result of a single comprehensive report documenting 12 years of dengue importation to China. Secondly, the study only used data with defined laboratory confirmed cases of dengue, or probable cases aligned with the U.S. CDC’s 2015 criteria. Given the plethora of cases reported and defined by the articles, the classification of confirmed case legitimacy largely depended on the original authors’ discretion. Usage of laboratory confirmed cases is critical in this review. However, this had resulted in an exclusion of 11% of articles from the secondary screen, which potentially documented significant number of cases collectively. The employment of this case classification meant the consolidation of only a baseline number of cases representing the tip of the iceberg of dengue importation worldwide. Wealthier countries with the resources and capacity to conduct laboratory testing and importation surveillance would also have been over-represented. Third, dengue importation is directly affected by tourism preference in travellers, as seen with the example of Myanmar and Africa—both are areas of prolific dengue transmission with relatively less dengue importation due to a low tourism traffic. This indicates that dengue importation trends cannot be used as a standalone to predict dengue hotspots. Moreover, the traveling patterns of the international population can be dynamic, unpredictable and not well accountable. The trends observed in this study may not be generalizable to future trends. There lies a possibility that the highlighted countries may not be the only threats in the next decade. Lastly, there were about 50% of imported cases from this review with unreported origins due to the limited data presented in available literature. Knowledge of these sources may change the trends of the sources of imported dengue globally. This also highlights the importance of reporting the travel destinations for future studies focusing on dengue importation.

## Conclusion

Dengue is an emerging global health concern that requires more attention from not only dengue endemic countries, but also increasingly from non-dengue endemic countries. With globalisation and global warming enabling prolific establishment of *Aedes* and transport of viremic travellers, the threat of dengue seeding globally is emerging. Dengue endemic nation with high influx of travellers should prioritize prevention and mitigation measures to reduce dengue activity, and non-dengue endemic countries should strengthen travel advice focusing on appropriate preventive measures for their residents traveling to dengue endemic countries. In addition, political commitment and sustainable resources are critical to enhance environmental surveillance to monitor and control vector population as well as to strengthen laboratory diagnosis capability to detect dengue early for prompt responses. This study should serve as quantitative evidence indicative of the burgeoning threat of dengue to global health security, to guide policy and intervention-decision process.

## Supplementary Information

**Additional file 1.** Search Query Strings, Additional Methodological Details, Specific Country Sources of Importation to US and Europe, and Additional References.

## Data Availability

All data relevant to the study are included in the article or uploaded as Additional information.

## Abbreviations

CDC: Central for Disease Prevention and Control
COVID-19: Coronavirus disease 2019
DENV: Dengue virus
PRISMA: Preferred Reporting Items for Systematic Reviews and Meta-Analyses
RT-PCR: Reverse transcription polymerase chain reaction
SARS-CoV-2: Severe acute respiratory syndrome coronavirus 2
UN: United Nation
US: United States
WHO: World Health Organisation
