# Supplementary material for: Global dengue importation: a systematic review
Source: BMC Infect Dis. 2021 Oct 19;21:1078. doi: 10.1186/s12879-021-06740-1 (PMC8524397; doi:10.1186/s12879-021-06740-1)
Supplement: Supplementary file 1 — Additional file 1. Search Query Strings, Additional Methodological Details, Specific Country Sources of Importation to US and Europe, and Additional References. [file 12879_2021_6740_MOESM1_ESM.docx]

**Supplementary Material**

### **Query Strings Used**

**PubMed**

1533 items found

(((traveler OR travel OR imported))) AND ((dengue OR dengue case OR dengue outbreak))

**EMBASE**

1159 items found

imported AND 'dengue'/exp OR ((traveler OR travel OR imported) NEAR/15 dengue)

(imported dengue)/exp OR ((traveler OR travel OR imported) NEAR/15 dengue)

**Scopus**

988 items found

TITLE-ABS-KEY ( ( traveler  OR  travel  OR  imported )  W/15  dengue )

**Cochrane**

82 items found

“Imported dengue”

**Other Sources**

82 Articles

**Inclusion Criteria & Agenda**

Articles regarding imported dengue were consolidated from online databases PubMed, Scopus and EMBASE. The agenda was to compile imported dengue cases, defined as dengue illness contracted by individuals during their travel.

Articles screened had multiple classifications of dengue cases, including “reported cases”, “diagnosed cases”, “notified cases”, “probable cases”, “confirmed cases” and “laboratory confirmed cases”. Understanding and consistency of cases across articles was poor as some authors clearly defined cases included in their report while some did not. Definition of cases labelled identically also differed across articles, i.e. “probable cases” could entail cases with serological confirmation, or just cases with clinical diagnosis and relevant travel history. “Laboratory confirmed cases” could entail diagnosis via RT-PCR/NS1/Virus Isolation, or it could represent serological confirmation.

In view of above mentioned inconsistencies, cases labelled “laboratory confirmed cases” were included in our analysis. Cases were included regardless of their mode of confirmation (serology, protein, DNA or virus isolation), so long as authors defined the confirmation mode, respecting the articles’ author’s judgement of them being the most validated cases amongst all their cases.

There were many instances in which “probable cases” were grouped together with “laboratory confirmed cases”, reporting them as a single figure. “Probable cases” in these context were often confirmed by serological measures or a clinical diagnosis accompanied by weak serological confirmation. These “laboratory confirmed cases” were accepted as well.

Exceptions were made to include several probable cases that fitted CDC’s criteria for positive case confirmation or that were defined as serologically confirmed cases. The CDC criteria referenced for our inclusion can be found in the link below.

<https://wwwn.cdc.gov/nndss/conditions/dengue-virus-infections/case-definition/2015/>

The papers excluded under “others” in the PRISMA flowchart detailed dengue cases as percentages, which did not tally when back calculated.

**Data Cleaning Procedure**

**Full Dataset**

The following types of article were excluded from our study: Narrative reviews, conference proceedings, manuscript, letters, correspondences and articles in languages other than English and Chinese.

Fields extracted from articles include “Title”, “Author”, “Year of study”, “Country of Publication”, “Cases with vague case confirmation”, “Confirmed cases”, “Mode of diagnosis”, “Country and state imported to”, “Country and state imported from”, “Serotype” and “Related Outbreaks”.

Following data extraction, “Mode of diagnosis”, “Serotype” and “Related Outbreaks” data were dropped from the analysis due to incomplete and insignificant data collected from the articles, rendering a substantial analysis impossible.

Cleaning of extracted data constituted 3 main stages; exclusion by case type, dubious cases, and overlapping cases within countries.

In view of the varying case definitions employed by the authors. Cases included were strictly laboratory confirmed cases (as defined by the authors of the respective articles) and probable cases in some papers, whereby the probable cases defined was aligned with the definition of a positive case defined by CDC in 2015, or serologically confirmed.

Next, the following types of cases were further excluded as part of our exclusion criteria: Repeated cases mentioned by authors across articles (if identifiable), cases recording seroprevalence with unknown or farfetched travel history, unclear cases presented in percentages or proportions, and cases imported within the country.

Lastly, the following steps were taken in an attempt to remove repeated data. Data was sorted in accordance to countries dengue cases were imported to. Identical cases reported from the same country across articles were removed if the articles’ description of case details were the same. Timelines were constructed for each country to compare the location within which cases were reported from and time period in which cases were reported. Data from articles were taken in whole or in parts to form the most comprehensive timeline of imported dengue cases possible, without overlaps occurring in the country over the years. The article providing the widest timeframe across the biggest area was usually prioritized, followed by including articles with smaller timeframe or scale to supplement gaps in the timeline as much as possible.

Many articles presented their data in the following 2 formats: stratification of cases by year without mention of importation source, stratification of cases by importation source (region or country) over a range of years without mention of specific year of import. In view of our agenda to identify hotspots of dengue importation, our article prioritized the second presentation which emphasized geographical data, retaining data of cases imported over a known range of years with details on import source.

Data was cleaned to standardize all geographical data to state and country level. Data detailing cities were converted to the state in which they were located, and state data was converted to country level data. An additional field was created to classify location data regionally to allow the allocation of each case into specific regional source.

We followed the UN Geoscheme to group the countries into geographical regions, allowing regional comparison of dengue contributing sources. The classification scheme was selected based on its compatibility with majority of our data obtained from available literature, whereby origin of imported cases were often classified as Africa, Americas and Asia, instead of Asia-Pacific in the WHO regions scheme. Detailed list of countries classified under the respective regions can be found in the document (UN Geoscheme) appended.

**US Dataset**

The steps aforementioned were applied to the US dataset similarly.

Due to limitations of certain articles in presenting cases in distinct, single years, as some articles presented their cases in a range of years, temporal analysis of dengue cases imported to the US was only done in decades as we found it to be the year range in which cases could be well separated in and encompass all cases included from available literature.

**Data Analysis**

**Full Dataset**

The number of cases attributed to each region and its specific countries were tabulated, with specific identification of the top countries (hotspots) in each. No temporal analysis was conducted for imported cases reported globally. All data tables and charts are created using Microsoft Excel 2016.

Mapping of importation sources only utilized cases with importation region available at least. Such cases also included those associated with travel to multiple countries in the same region, which were replaced with a single region and retained in the analysis. Cases associated with travel to multiple regions and unknown locations were left out of the mapping. Maps were created to illustrate dengue importation sources at both regional level and also country-specific level.

**US Dataset**

The same workflow was applied to the US data; analysis and mapping of cases according to import regions and countries. From the available data, sum of cases reported from each US state was also tabulated and mapped. Following which, cases from the top reporting states, New York and Texas were analyzed for their importation origins.

US data was also stratified according to years in decades to visualize temporal trends of dengue imported into US.

**Europe dataset**

The same workflow was applied to the Europe data; analysis and mapping of cases according to import regions and countries. Sum of cases reported from each European country was also tabulated.

**Table 1:** Source of dengue cases imported globally (stratified by region and country)

| **Region/Country** | **Cases** |
| --- | --- |
| **Africa** | **883** |
| Angola | 245 |
| Benin | 15 |
| Botswana, South Africa & Zimbabwe | 1 |
| Burkina Faso | 12 |
| Burkina Faso & Mali | 2 |
| Cameroon | 7 |
| Central African Republic | 1 |
| Comoros | 12 |
| Democratic Republic of Congo | 5 |
| Djibouti | 5 |
| Egypt | 1 |
| Equatorial Guinea | 3 |
| Eritrea | 5 |
| Ethiopia | 1 |
| Gabon | 1 |
| Gambia | 3 |
| Ghana | 7 |
| Guinea | 1 |
| Ivory Coast | 15 |
| Kenya | 7 |
| Kenya & Tanzania | 1 |
| Madagascar | 4 |
| Mali | 1 |
| Mauritius | 2 |
| Mayotte | 2 |
| Morocco | 5 |
| Mozambique | 1 |
| Niger | 1 |
| Nigeria | 8 |
| Rwanda | 2 |
| Senegal | 6 |
| Seychelles | 12 |
| Somalia | 68 |
| South Africa | 5 |
| Tanzania | 12 |
| Togo | 2 |
| Uganda | 3 |
| Zambia | 2 |
| Zimbabwe | 1 |
| Unspecified Country in Africa | 396 |
| **Americas** | **2357** |
| Anguilla | 2 |
| Antilles & St. Martin | 1 |
| Argentina | 18 |
| Argentina, Bolivia, Brazil, Chile & Uruguay | 1 |
| Argentina, Brazil, Barbados, Chile, Columbia, Ecuador & Peru | 1 |
| Aruba | 4 |
| Aruba & Venezuela | 1 |
| Bahamas | 1 |
| Barbados | 22 |
| Belize | 5 |
| Bolivia | 9 |
| Brazil | 145 |
| British Virgin Islands | 24 |
| British Virgin Islands & Puerto Rico | 1 |
| British Virgin Islands & United States Virgin Islands | 1 |
| British Virgin Islands, Puerto Rico & United States Virgin Islands | 1 |
| Caribbean | 211 |
| Colombia | 21 |
| Costa Rica | 49 |
| Cuba | 9 |
| Curacao | 5 |
| Dominica | 1 |
| Dominican Republic | 81 |
| Dominican Republic & Puerto Rico | 1 |
| Dutch Antilles | 2 |
| Ecuador | 9 |
| El Salvado & Mexico | 1 |
| El Salvador | 27 |
| El Salvador, Guatemala & Mexico | 1 |
| French Guiana | 50 |
| French West Indies | 2 |
| French West Indies & Puerto Rico | 1 |
| Grenada | 2 |
| Grenadine Islands | 2 |
| Greneda | 1 |
| Greneda & Puerto Rico | 2 |
| Guadeloupe | 77 |
| Guadeloupe & Martinique | 1 |
| Guatemala | 20 |
| Guatemala & Honduras | 1 |
| Guatemala & Mexico | 1 |
| Guyana | 1 |
| Haiti | 101 |
| Haiti & Turks and Caicos Islands | 1 |
| Hawaii | 4 |
| Honduras | 29 |
| Jamaica | 33 |
| Martinique | 59 |
| Mexico | 215 |
| Nicaragua | 40 |
| Panama | 6 |
| Paraguay | 38 |
| Paraguay, Brazil, Costa Rica, Venezuela | 45 |
| Peru | 5 |
| Puerto Rico | 92 |
| Puerto Rico & St. Croix | 1 |
| Puerto Rico & US Virgin Islands | 1 |
| St. Barthelemy | 4 |
| St. Barthelemy & St. Thomas | 1 |
| St. Bartholomew | 1 |
| St. Croix | 3 |
| St. John | 1 |
| St. Kitt's | 1 |
| St. Lucia | 4 |
| St. Martin | 3 |
| St. Thomas | 10 |
| St. Vincent | 1 |
| Suriname | 8 |
| Tanzania | 6 |
| Tobago | 1 |
| Tortola | 16 |
| Trinidad | 4 |
| Trinidad & Tobago | 1 |
| Trinidad & Venezuela | 1 |
| United States Virgin Islands | 16 |
| Venezuela | 31 |
| Virgin Islands | 8 |
| West Indies | 2 |
| Unspecified Country in Americas | 376 |
| Unspecified Country in Central America | 117 |
| Unspecified Country in Latin America | 63 |
| Unspecified Country in Middle America | 3 |
| Unspecified Country in North America | 60 |
| Unspecified Country in South America | 129 |
| **Asia** | **11421** |
| Bangladesh | 90 |
| Bangladesh & India | 20 |
| Borneo | 3 |
| Cambodia | 146 |
| Cambodia & Thailand | 2 |
| Cambodia, Laos, Myanmar & Thailand | 1 |
| Cambodia, Malaysia, Philippines & Thailand | 1 |
| Cambodia, India, Laos, Thailand & Vietnam | 35 |
| China | 31 |
| China & Hong Kong | 2 |
| China, India & Nepal | 1 |
| East Timor | 55 |
| Hong Kong & Philippines | 1 |
| Hong Kong, Japan & Thailand | 1 |
| Hong Kong, Laos & Thailand | 1 |
| India | 741 |
| India & Nepal | 1 |
| India & Pakistan | 2 |
| India & Philippines | 1 |
| India & Saudi Arabia | 1 |
| India & Sri Lanka | 2 |
| India & Thailand | 1 |
| India & Vietnam | 1 |
| India, Malaysia & Singapore | 1 |
| India, Nepal & Thailand | 2 |
| India, Thailand & United Arab Emirates | 1 |
| Indian Subcontinent | 100 |
| Indonesia | 1449 |
| Indonesia & Thailand | 1 |
| Indonesia & Singapore | 2 |
| Japan | 5 |
| Laos | 41 |
| Malaysia | 417 |
| Maldives | 74 |
| Myanmar | 1505 |
| Myanmar & Thailand | 1 |
| Nepal | 8 |
| Pakistan | 16 |
| Philippines | 876 |
| Philippines & Thailand | 1 |
| Saudi Arabia | 14 |
| Singapore | 103 |
| Singapore & Thailand | 1 |
| Sri Lanka | 136 |
| Sudan | 1 |
| Taiwan | 8 |
| Thailand | 2088 |
| Vietnam | 309 |
| Vietnam & Thailand | 4 |
| Yemen | 5 |
| Unspecified Country in Asia | 3112 |
| **Europe** | **14** |
| Portugal | 8 |
| Unspecified Country in Europe | 6 |
| **Multiple** | **23** |
| Asia & Micronesia | 1 |
| Australia & Southeast Asia | 1 |
| Australia & Thailand | 1 |
| Cambodia, Kenya & Somalia | 1 |
| China, Egypt & Ethiopia | 1 |
| Haiti & Zambia | 1 |
| Multiple Unknown Regions | 17 |
| **Oceania** | **297** |
| Australia | 6 |
| Australia, Fiji & New Zealand | 1 |
| Australia, Fiji, New Zealand & Tahiti | 1 |
| Australia, New Zealand & Tahiti | 2 |
| Cook Islands | 5 |
| Fiji | 18 |
| Fiji, New Zealand & Tahiti | 1 |
| French Polynesia | 16 |
| French Polynesia & New Caledonia | 7 |
| Guam | 2 |
| Marshall Islands | 2 |
| Moorea Island | 1 |
| Nauru | 2 |
| New Caledonia | 6 |
| New Guinea | 1 |
| New Zealand | 4 |
| Pacific Islands | 15 |
| Palau | 1 |
| Papua New Guinea | 62 |
| Pitcairn Island | 1 |
| Samoa | 10 |
| Solomon Islands | 5 |
| Tahiti | 17 |
| Tonga | 4 |
| Tuvalu | 4 |
| Vanuatu | 5 |
| Unspecified Country in Oceania | 98 |
| **Unknown** | **15410** |
| **Grand Total** | **30405** |

**Table 2:** Sources of dengue importation to US (stratified by region and country)

| **Region/Country** | **Sum of Cases** |
| --- | --- |
| **Africa** | **102** |
| Botswana, Zimbabwe & South African Regions | 1 |
| Eritrea | 1 |
| Ethiopia | 1 |
| Ghana | 1 |
| Ivory Coast | 1 |
| Kenya | 1 |
| Kenya & Tanzania | 1 |
| Madagascar | 1 |
| Niger | 1 |
| Nigeria | 4 |
| Senegal | 1 |
| Somalia | 62 |
| South Africa | 1 |
| Unspecified Country in Africa | 25 |
| **Americas** | **846** |
| Anguilla | 2 |
| Antilles & St. Martin | 1 |
| Argentina, Barbados, Brazil, Chile, Columbia, Ecuador & Peru | 1 |
| Aruba & Venezuela | 1 |
| Bahamas | 1 |
| Barbados | 11 |
| Belize | 4 |
| Brazil | 14 |
| British Virgin Islands | 9 |
| British Virgin Islands & Puerto Rico | 1 |
| British Virgin Islands & United States Virgin Islands | 1 |
| British Virgin Islands, Puerto Rico & United States Virgin Islands | 1 |
| Calcos, Haiti & Turks | 1 |
| Caribbean | 49 |
| Colombia | 7 |
| Costa Rica | 16 |
| Dominican Republic | 57 |
| Dominican Republic & Puerto Rico | 1 |
| Ecuador | 6 |
| El Salvador | 20 |
| El Salvador & Mexico | 1 |
| El Salvador, Guatemala & Mexico | 1 |
| French West Indies | 1 |
| French West Indies & Puerto Rico | 1 |
| Grenada | 2 |
| Grenadine Islands | 2 |
| Greneda & Puerto Rico | 2 |
| Guatemala | 13 |
| Guatemala & Honduras | 1 |
| Guatemala & Mexico | 1 |
| Guyana | 1 |
| Haiti | 68 |
| Hawaii | 2 |
| Honduras | 25 |
| Jamaica | 20 |
| Mexico | 197 |
| Nicaragua | 30 |
| Panama | 4 |
| Paraguay | 2 |
| Peru | 3 |
| Puerto Rico | 89 |
| Puerto Rico & St. Croix | 1 |
| Puerto Rico & United States Virgin Islands | 1 |
| St. Barthelemy | 4 |
| St. Barthelemy & St. Thomas | 1 |
| St. Bartholomew | 1 |
| St. Croix | 3 |
| St. Croix & United States Virgin Islands | 1 |
| St. John | 1 |
| St. Kitt's | 1 |
| St. Lucia | 3 |
| St. Martin | 2 |
| St. Thomas | 10 |
| St. Vincent | 1 |
| Tortola | 16 |
| Trinidad | 4 |
| Trinidad & Tobago | 1 |
| Trinidad & Venezuela | 1 |
| United States Virgin Islands | 13 |
| Venezuela | 6 |
| Virgin Islands | 8 |
| Unspecified Country in Americas | 16 |
| Unspecified Country in Central America | 18 |
| Unspecified Country in North America | 53 |
| Unspecified Country in South America | 10 |
| **Asia** | **531** |
| Bangladesh | 13 |
| Cambodia | 1 |
| Cambodia, Laos, Myanmar & Thailand | 1 |
| China, India & Nepal | 1 |
| East Timor | 1 |
| Hong Kong & Philippines | 1 |
| Hong Kong, Japan & Thailand | 1 |
| Hong Kong, Laos & Thailand | 1 |
| India | 44 |
| India & Philippines | 1 |
| India & Sri Lanka | 1 |
| India & Vietnam | 1 |
| India, Nepal & Thailand | 2 |
| Indonesia | 14 |
| Japan | 2 |
| Laos | 1 |
| Malaysia | 3 |
| Maldives | 1 |
| Myanmar | 2 |
| Myanmar & Thailand | 1 |
| Nepal | 1 |
| Pakistan | 1 |
| Philippines | 77 |
| Philippines & Thailand | 1 |
| Saudi Arabia | 1 |
| Singapore | 4 |
| Sri Lanka | 5 |
| Taiwan | 2 |
| Thailand | 40 |
| Vietnam | 3 |
| Unspecified Country in Asia | 303 |
| **Multiple** | **6** |
| Asia & Micronesia | 1 |
| Australia & Southeast Asia | 1 |
| Australia & Thailand | 1 |
| Cambodia, Kenya & Somalia | 1 |
| China, Egypt & Ethiopia | 1 |
| Haiti & Zambia | 1 |
| **Oceania** | **50** |
| Australia | 1 |
| Australia, Fiji & New Zealand | 1 |
| Australia, Fiji, New Zealand & Tahiti | 1 |
| Australia, New Zealand & Tahiti | 2 |
| Fiji | 4 |
| Fiji, New Zealand & Tahiti | 1 |
| French Polynesia | 2 |
| Marshall Islands | 1 |
| New Guinea | 1 |
| New Zealand | 1 |
| Samoa | 2 |
| Tahiti | 12 |
| Tonga | 1 |
| Unspecified Country in Oceania | 20 |
| **Unknown** | **941** |
| **Grand Total** | **2476** |

**Table 3: Imported dengue cases reported by various US states**

| **State** | **Sum of Cases** |
| --- | --- |
| Alabama | 9 |
| Alaska | 5 |
| Arizona | 96 |
| Arkansas | 2 |
| California | 155 |
| Colorado | 21 |
| Connecticut | 16 |
| Delaware | 4 |
| District of Columbia | 19 |
| Florida | 166 |
| Georgia | 51 |
| Hawaii | 59 |
| Idaho | 2 |
| Illinois | 46 |
| Indiana | 4 |
| Iowa | 21 |
| Kansas | 4 |
| Kentucky | 1 |
| Louisiana | 2 |
| Maine | 13 |
| Maryland | 36 |
| Massachusetts | 129 |
| Michigan | 25 |
| Minnesota | 67 |
| Misouri | 1 |
| Mississippi | 3 |
| Missouri | 20 |
| Montana | 1 |
| Nebraska | 2 |
| Nevada | 8 |
| New Hampshire | 5 |
| New Jersey | 32 |
| New Mexico | 4 |
| New York | 379 |
| North Carolina | 28 |
| North Dakota | 1 |
| Ohio | 52 |
| Oklahoma | 9 |
| Oregon | 27 |
| Pennsylvania | 37 |
| Rhode Island | 14 |
| South Carolina | 2 |
| South Dakota | 5 |
| Tennessee | 2 |
| Texas | 260 |
| Utah | 8 |
| Vermont | 5 |
| Virginia | 51 |
| Washington | 66 |
| Wisconsin | 59 |
| Wyoming | 3 |
| Multiple States | 19 |
| Unknown | 420 |
| **Grand Total** | **2476** |

**Table 4:** Imported dengue cases reported by New York

| **Country** | **Sum of Cases** |
| --- | --- |
| Anguilla | 1 |
| Antilles & St. Martin | 1 |
| Bangladesh | 3 |
| Barbados | 2 |
| British Virgin Islands & United States Virgin Islands | 1 |
| Burma | 1 |
| Caribbean | 2 |
| Colombia | 2 |
| Dominican Republic | 26 |
| East Timor | 1 |
| El Salvador | 2 |
| Grenada | 1 |
| Guatemala | 6 |
| Haiti | 4 |
| Haiti & Zambia | 1 |
| Honduras | 2 |
| Hong Kong, Laos & Thailand | 1 |
| India | 3 |
| Indonesia | 3 |
| Jamaica | 4 |
| Mexico | 1 |
| Myanmar | 1 |
| Nicaragua | 11 |
| Nigeria | 1 |
| Peru | 1 |
| Philippines | 4 |
| Puerto Rico | 23 |
| Singapore | 1 |
| Somalia | 1 |
| South Africa | 1 |
| St. Martin | 1 |
| St. Thomas | 1 |
| Thailand | 6 |
| Tortola | 3 |
| Trinidad & Tobago | 1 |
| Trinidad & Venezuela | 1 |
| United States Virgin Islands | 2 |
| Vietnam | 1 |
| Virgin Islands | 2 |
| Unknown | 249 |
| **Grand Total** | **379** |

**Table 5:** Imported dengue cases reported by Texas

| **Country** | **Sum of Cases** |
| --- | --- |
| Bangladesh | 3 |
| Belize | 1 |
| Brazil | 2 |
| Caribbean | 5 |
| Costa Rica | 1 |
| El Salvador | 5 |
| El Salvador & Mexico | 1 |
| El Salvador, Guatemala & Mexico | 1 |
| Grenada & Puerto Rico | 2 |
| Guatemala | 3 |
| Honduras | 8 |
| India | 6 |
| Malaysia | 1 |
| Maldives | 1 |
| Mexico | 105 |
| Nicaragua | 2 |
| Nigeria | 1 |
| Pakistan | 1 |
| Panama | 1 |
| Paraguay | 1 |
| Philippines | 1 |
| Puerto Rico | 6 |
| Singapore | 2 |
| St. Croix | 1 |
| Tahiti | 2 |
| Thailand | 1 |
| Tortola | 1 |
| Venezuela | 2 |
| Unknown | 93 |
| **Grand Total** | **260** |

**Table 6:** Dengue cases imported to the US [Stratified by time period (decades) and source (region only)]

| **Cases** | **Period** | | | | | |
| --- | --- | --- | --- | --- | --- | --- |
| **Region** | **1977 - 1980** | **1981 - 1990** | **1991 - 2000** | **2001 - 2010** | **2011 - 2017** | **Total** |
| Africa |  | 7 | 66 | 8 | 21 | 102 |
| Americas | 20 | 63 | 302 | 252 | 209 | 846 |
| Asia | 1 | 82 | 91 | 73 | 284 | 531 |
| Multiple |  | 1 | 3 | 2 |  | 6 |
| Oceania |  | 12 | 7 | 11 | 20 | 50 |
| Unknown | 118 | 131 | 55 | 614 | 23 | 941 |
| **Grand Total** | **139** | **296** | **524** | **960** | **557** | **2476** |

**Table 7:** Dengue cases imported to the US [stratified by time period (decades) and source (region and country)]

|  | **Period** | | | | | |
| --- | --- | --- | --- | --- | --- | --- |
| **Region/Country** | **1977 - 1980** | **1981 - 1990** | **1991 - 2000** | **2001 - 2010** | **2011 - 2017** | **Total** |
| **Africa** |  | **7** | **66** | **8** | **21** | **102** |
| Botswana, Zimbabwe & South African Regions |  |  |  | 1 |  | 1 |
| Eritrea |  |  | 1 |  |  | 1 |
| Ethiopia |  |  | 1 |  |  | 1 |
| Ghana |  |  |  | 1 |  | 1 |
| Ivory Coast |  |  | 1 |  |  | 1 |
| Kenya |  | 1 |  |  |  | 1 |
| Kenya & Tanzania |  | 1 |  |  |  | 1 |
| Madagascar |  | 1 |  |  |  | 1 |
| Niger |  |  | 1 |  |  | 1 |
| Nigeria |  | 1 | 1 | 2 |  | 4 |
| Senegal |  |  | 1 |  |  | 1 |
| Somalia |  | 3 | 59 |  |  | 62 |
| South Africa |  |  | 1 |  |  | 1 |
| Unspecified Country in Africa |  |  |  | 4 | 21 | 25 |
| **Americas** | **20** | **63** | **302** | **252** | **209** | **846** |
| Anguilla |  |  | 2 |  |  | 2 |
| Antilles & St. Martin |  |  |  | 1 |  | 1 |
| Argentina, Barbados, Brazil, Chile, Columbia, Ecuador & Peru |  |  |  | 1 |  | 1 |
| Aruba & Venezuela |  |  | 1 |  |  | 1 |
| Bahamas |  |  | 1 |  |  | 1 |
| Barbados |  |  | 10 | 1 |  | 11 |
| Belize |  |  | 3 | 1 |  | 4 |
| Brazil |  |  | 2 | 12 |  | 14 |
| British Virgin Islands |  |  | 9 |  |  | 9 |
| British Virgin Islands & Puerto Rico |  |  | 1 |  |  | 1 |
| British Virgin Islands & United States Virgin Islands |  |  | 1 |  |  | 1 |
| British Virgin Islands, Puerto Rico & United States Virgin Islands |  |  | 1 |  |  | 1 |
| Calcos, Haiti & Turks |  |  |  | 1 |  | 1 |
| Caribbean |  | 5 | 13 | 2 | 29 | 49 |
| Colombia |  | 2 | 5 |  |  | 7 |
| Costa Rica |  |  | 2 | 14 |  | 16 |
| Dominican Republic |  | 1 | 12 | 37 | 7 | 57 |
| Dominican Republic & Puerto Rico |  |  | 1 |  |  | 1 |
| Ecuador |  |  | 3 | 2 | 1 | 6 |
| El Salvador |  | 1 | 11 | 7 | 1 | 20 |
| El Salvador & Mexico |  |  | 1 |  |  | 1 |
| El Salvador, Guatemala & Mexico |  |  | 1 |  |  | 1 |
| French West Indies |  | 1 |  |  |  | 1 |
| French West Indies & Puerto Rico |  |  | 1 |  |  | 1 |
| Grenada |  |  | 1 | 1 |  | 2 |
| Grenadine Islands |  |  | 2 |  |  | 2 |
| Greneda & Puerto Rico |  |  | 2 |  |  | 2 |
| Guatemala |  |  | 6 | 7 |  | 13 |
| Guatemala & Honduras |  |  |  | 1 |  | 1 |
| Guatemala & Mexico |  |  |  | 1 |  | 1 |
| Guyana |  |  |  | 1 |  | 1 |
| Haiti |  | 5 | 50 | 11 | 2 | 68 |
| Hawaii |  |  |  | 2 |  | 2 |
| Honduras |  |  | 15 | 10 |  | 25 |
| Jamaica |  | 6 | 8 | 2 | 4 | 20 |
| Mexico | 20 | 8 | 36 | 50 | 83 | 197 |
| Nicaragua |  | 1 | 8 | 20 | 1 | 30 |
| Panama |  |  | 3 | 1 |  | 4 |
| Paraguay |  | 1 |  | 1 |  | 2 |
| Peru |  | 1 |  | 2 |  | 3 |
| Puerto Rico |  | 10 | 43 | 36 |  | 89 |
| Puerto Rico & St. Croix |  |  | 1 |  |  | 1 |
| Puerto Rico & United States Virgin Islands |  |  | 1 |  |  | 1 |
| St. Barthelemy |  |  |  | 4 |  | 4 |
| St. Barthelemy & St. Thomas |  |  |  | 1 |  | 1 |
| St. Bartholomew |  |  | 1 |  |  | 1 |
| St. Croix |  |  | 2 | 1 |  | 3 |
| St. Croix & United States Virgin Islands |  | 1 |  |  |  | 1 |
| St. John |  |  | 1 |  |  | 1 |
| St. Kitt's |  |  |  | 1 |  | 1 |
| St. Lucia |  | 1 |  | 2 |  | 3 |
| St. Martin |  |  | 1 | 1 |  | 2 |
| St. Thomas |  |  | 9 | 1 |  | 10 |
| St. Vincent |  | 1 |  |  |  | 1 |
| Tortola |  |  | 16 |  |  | 16 |
| Trinidad |  | 1 | 1 | 2 |  | 4 |
| Trinidad & Tobago |  |  | 1 |  |  | 1 |
| Trinidad & Venezuela |  | 1 |  |  |  | 1 |
| United States Virgin Islands |  | 8 | 3 | 2 |  | 13 |
| Venezuela |  | 1 | 2 | 3 |  | 6 |
| Virgin Islands |  | 5 | 1 | 2 |  | 8 |
| Unspecified Country in Americas |  | 2 | 7 | 7 |  | 16 |
| Unspecified Country in Central America |  |  |  |  | 18 | 18 |
| Unspecified Country in North America |  |  |  |  | 53 | 53 |
| Unspecified Country in South America |  |  |  |  | 10 | 10 |
| **Asia** | **1** | **82** | **91** | **73** | **284** | **531** |
| Bangladesh |  | 1 | 6 | 6 |  | 13 |
| Cambodia |  |  |  | 1 |  | 1 |
| Cambodia, Laos, Myanmar & Thailand |  |  |  | 1 |  | 1 |
| China, India & Nepal |  |  |  | 1 |  | 1 |
| East Timor |  |  | 1 |  |  | 1 |
| Hong Kong & Philippines |  |  | 1 |  |  | 1 |
| Hong Kong, Japan & Thailand |  | 1 |  |  |  | 1 |
| Hong Kong, Laos & Thailand |  | 1 |  |  |  | 1 |
| India | 1 | 8 | 13 | 22 |  | 44 |
| India & Philippines |  |  | 1 |  |  | 1 |
| India & Sri Lanka |  | 1 |  |  |  | 1 |
| India & Vietnam |  |  |  | 1 |  | 1 |
| India, Nepal & Thailand |  |  | 1 | 1 |  | 2 |
| Indonesia |  | 2 | 7 | 4 | 1 | 14 |
| Japan |  |  | 2 |  |  | 2 |
| Laos |  |  |  | 1 |  | 1 |
| Malaysia |  |  | 2 | 1 |  | 3 |
| Maldives |  |  |  | 1 |  | 1 |
| Myanmar |  |  | 2 |  |  | 2 |
| Myanmar & Thailand |  |  | 1 |  |  | 1 |
| Nepal |  | 1 |  |  |  | 1 |
| Pakistan |  |  |  | 1 |  | 1 |
| Philippines |  | 56 | 13 | 8 |  | 77 |
| Philippines & Thailand |  |  | 1 |  |  | 1 |
| Saudi Arabia |  |  |  | 1 |  | 1 |
| Singapore |  |  | 1 | 3 |  | 4 |
| Sri Lanka |  | 1 | 1 | 2 | 1 | 5 |
| Taiwan |  | 1 | 1 |  |  | 2 |
| Thailand |  | 5 | 22 | 13 |  | 40 |
| Vietnam |  |  | 3 |  |  | 3 |
| Unspecified Country in Asia |  | 4 | 12 | 5 | 282 | 303 |
| **Multiple** |  | **1** | **3** | **2** |  | **6** |
| Asia & Micronesia |  | 1 |  |  |  | 1 |
| Australia & Southeast Asia |  |  | 1 |  |  | 1 |
| Australia & Thailand |  |  | 1 |  |  | 1 |
| Cambodia, Kenya & Somalia |  |  | 1 |  |  | 1 |
| China, Egypt & Ethiopia |  |  |  | 1 |  | 1 |
| Haiti & Zambia |  |  |  | 1 |  | 1 |
| **Oceania** |  | **12** | **7** | **11** | **20** | **50** |
| Australia |  |  |  | 1 |  | 1 |
| Australia, Fiji & New Zealand |  | 1 |  |  |  | 1 |
| Australia, Fiji, New Zealand & Tahiti |  | 1 |  |  |  | 1 |
| Australia, New Zealand & Tahiti |  | 2 |  |  |  | 2 |
| Fiji |  | 3 |  | 1 |  | 4 |
| Fiji, New Zealand & Tahiti |  | 1 |  |  |  | 1 |
| French Polynesia |  |  |  | 2 |  | 2 |
| Marshall Islands |  |  |  | 1 |  | 1 |
| New Guinea |  | 1 |  |  |  | 1 |
| New Zealand |  |  |  | 1 |  | 1 |
| Samoa |  |  | 2 |  |  | 2 |
| Tahiti |  | 3 | 4 | 5 |  | 12 |
| Tonga |  |  | 1 |  |  | 1 |
| Unspecified Country in Oceania |  |  |  |  | 20 | 20 |
| **Unknown** | **118** | **131** | **55** | **614** | **23** | **941** |
| **Grand Total** | **139** | **296** | **524** | **960** | **557** | **2476** |

**Table 8:** Dengue cases reported by European countries

| **Country in Europe** | **Cases** |
| --- | --- |
| Austria | 95 |
| Belgium | 652 |
| Czech Republic | 150 |
| Denmark | 195 |
| Europe | 1724 |
| Finland | 116 |
| France | 568 |
| Germany | 723 |
| Italy | 240 |
| Netherlands | 100 |
| Norway | 9 |
| Poland | 215 |
| Portugal | 148 |
| Romania | 15 |
| Russia | 249 |
| Serbia | 1 |
| Spain | 602 |
| Sweden | 1109 |
| Switzerland | 72 |
| United Kingdom | 87 |
| **Grand Total** | **7070** |

**Table 9:** Dengue cases imported to Europe (stratified by region and country)

| **Region/Country** | **Cases** |
| --- | --- |
| **Africa** | **452** |
| Angola | 156 |
| Benin | 14 |
| Burkina Faso | 10 |
| Burkina Faso & Mali | 2 |
| Cameroon | 7 |
| Central African Republic | 1 |
| Comoros | 12 |
| Democratic Republic of Congo | 4 |
| Djibouti | 4 |
| Egypt | 1 |
| Equatorial Guinea | 3 |
| Eritrea | 4 |
| Gabon | 1 |
| Gambia | 3 |
| Ghana | 6 |
| Guinea | 1 |
| Ivory Coast | 11 |
| Kenya | 3 |
| Madagascar | 2 |
| Mauritius | 2 |
| Mayotte | 2 |
| Morocco | 3 |
| Mozambique | 1 |
| Nigeria | 2 |
| Rwanda | 1 |
| Senegal | 5 |
| Seychelles | 6 |
| Somalia | 4 |
| South Africa | 1 |
| Tanzania | 10 |
| Togo | 2 |
| Uganda | 2 |
| Zambia | 2 |
| Zimbabwe | 1 |
| Unspecified Country in Africa | 163 |
| **Americas** | **915** |
| Argentina, Bolivia, Brazil, Chile & Uruguay | 1 |
| Aruba | 4 |
| Barbados | 4 |
| Bolivia | 4 |
| Brazil | 72 |
| Caribbean | 46 |
| Colombia | 12 |
| Costa Rica | 31 |
| Cuba | 9 |
| Curacao | 5 |
| Dominican Republic | 24 |
| Dutch Antilles | 2 |
| Ecuador | 3 |
| El Salvador | 7 |
| French Guiana | 50 |
| French West Indies | 1 |
| Greneda | 1 |
| Guadeloupe | 77 |
| Guadeloupe & Martinique | 1 |
| Guatemala | 4 |
| Haiti | 5 |
| Honduras | 3 |
| Jamaica | 5 |
| Martinique | 59 |
| Mexico | 15 |
| Nicaragua | 9 |
| Panama | 1 |
| Paraguay | 2 |
| Puerto Rico | 3 |
| St. Martin | 1 |
| Suriname | 8 |
| Tobago | 1 |
| Venezuela | 24 |
| West Indies | 2 |
| United States Virgin Islands | 2 |
| Unspecified Country in Americas | 284 |
| Unspecified Country in Central America | 13 |
| Unspecified Country in Latin America | 62 |
| Unspecified Country in Middle America | 3 |
| Unspecified Country in South America | 55 |
| **Asia** | **2758** |
| Bangladesh | 35 |
| Bangladesh & India | 20 |
| Borneo | 2 |
| Cambodia | 19 |
| Cambodia, Malaysia, Philippines & Thailand | 1 |
| China | 1 |
| China & Hong Kong | 2 |
| East Timor | 1 |
| India | 275 |
| India & Nepal | 1 |
| India & Pakistan | 2 |
| India & Sri Lanka | 1 |
| India & Thailand | 1 |
| India, Thailand & United Arab Emirates | 1 |
| Indian Subcontinent | 23 |
| Indonesia | 303 |
| Indonesia & Singapore | 2 |
| Japan | 2 |
| Laos | 10 |
| Malaysia | 59 |
| Maldives | 43 |
| Myanmar | 12 |
| Nepal | 3 |
| Pakistan | 8 |
| Philippines | 69 |
| Saudi Arabia | 3 |
| Singapore | 2 |
| Singapore & Thailand | 1 |
| Sri Lanka | 56 |
| Thailand | 1044 |
| Vietnam | 28 |
| Vietnam & Thailand | 4 |
| Unspecified Country in Asia | 724 |
| **Europe** | **8** |
| Portugal | 8 |
| **Multiple** | **17** |
| Multiple Unknown Regions | 17 |
| **Oceania** | **44** |
| Cook Islands | 1 |
| Fiji | 5 |
| French Polynesia | 3 |
| French Polynesia & New Caledonia | 7 |
| New Caledonia | 3 |
| New Zealand | 3 |
| Pacific Islands | 7 |
| Papua New Guinea | 3 |
| Tahiti | 3 |
| Tonga | 1 |
| Unspecified Country in Oceania | 8 |
| **Unknown** | **2876** |
| **Grand Total** | **7070** |

**References**

1. Abdulsalam SM, Al-Tarrah MY, Alshalfan F: Dengue fever among travelers. 2012, 44:146-148.

2. Adams LE, Martin SW, Lindsey NP, Lehman JA, Rivera A, Kolsin J, Landry K, Staples JE, Sharp TM, Paz-Bailey G et al: Epidemiology of Dengue, Chikungunya, and Zika Virus Disease in U.S. States and Territories, 2017. Am J Trop Med Hyg 2019, 101(4):884-890.

3. Akter R, Naish S, Hu W, Tong S: Socio-demographic, ecological factors and dengue infection trends in Australia. PLoS One 2017, 12(10):e0185551.

4. Al Awaidy ST, Al Obeidani I, Bawikar S, Al Mahrouqi S, Al Busaidy SS, Al Baqlani S, Patel PK: Dengue epidemiological trend in Oman: a 13-year national surveillance and strategic proposition of imported cases. Trop Doct 2014, 44(4):190-195.

5. Al-Lawati ZH, Hariadi NI, Gilsdorf JR: Fever and abdominal pain in a 5-year-old traveler. Clin Pediatr (Phila) 2012, 51(6):602-605.

6. Alang N, Glavis-Bloom J, Alexander-Scott N, Mermel LA, Mileno MD: Surveillance of Travel-Related Mosquito-borne Illness in Rhode Island. R I Med J (2013) 2016, 99(7):22-23.

7. Allwinn R: Significant increase in travel-associated dengue fever in Germany. Med Microbiol Immunol 2011, 200(3):155-159.

8. Ansart S, Perez L, Vergely O, Danis M, Bricaire F, Caumes E: Illnesses in travelers returning from the tropics: a prospective study of 622 patients. J Travel Med 2005, 12(6):312-318.

9. Ansart Sv, Pajot O, Grivois J-P, Zeiler Vr, Klement E, Perez L, Bossi P, Bricaire F, Caumes E: Pneumonia among travelers returning from abroad. Journal of travel medicine 2004, 11(2):87-91.

10. Aubry M, Mapotoeke M, Teissier A, Paoaafaite T, Dumas-Chastang E, Giard M, Cao-Lormeau VM: Dengue virus serotype 2 (DENV-2) outbreak, French Polynesia, 2019. Euro Surveill 2019, 24(29).

11. Baaten GG, Sonder GJ, Zaaijer HL, van Gool T, Kint JA, van den Hoek A: Travel-related dengue virus infection, The Netherlands, 2006-2007. Emerg Infect Dis 2011, 17(5):821-828.

12. Badiaga S, Barrau K, Brouqui P, Durant J, Malvy D, Janbon F, Bonnet E, Bosseray A, Sotto A, Peyramont D et al: Imported Dengue in French University Hospitals: a 6-year survey. J Travel Med 2003, 10(5):286-289.

13. Bajjou T, Akhouad Y, Hilali F, Elkochri S, Laraqui A, Touil N, Amine IL, Mahassine F, Sekhsokh Y: Dengue fever in Morocco: result of surveillance during the year 2017 and first imported cases. International Journal of Research in Medical Sciences 2018, 6:1029-1032.

14. Bakker RC, Veenstra J, Dingemans-Dumas AM, Wetsteyn J, Kager PA: Imported Dengue in The Netherlands. J Travel Med 1996, 3(4):204-208.

15. Barrero PR, Mistchenko AS: Complete genome sequencing of dengue virus type 1 isolated in Buenos Aires, Argentina. Virus Res 2004, 101(2):135-145.

16. Barrero PR, Mistchenko AS: Genetic analysis of dengue virus type 3 isolated in Buenos Aires, Argentina. Virus Res 2008, 135(1):83-88.

17. Bellon MM, MacLean JD: A point source dengue outbreak in Canadian tourists in Barbados. Can Commun Dis Rep 1998, 24(20):161-164.

18. Blackburn NK, Meenehan G, Aldridge N: The status of dengue fever virus in South Africa — serological studies and diagnosis of a case of dengue fever. Transactions of The Royal Society of Tropical Medicine and Hygiene 1987, 81(4):690-692.

19. Blackburn NK, Rawat R: Dengue fever imported from India. A report of 3 cases. S Afr Med J 1987, 71(6):386-387.

20. Boggild AK, Esposito DH, Kozarsky PE, Ansdell V, Beeching NJ, Campion D, Castelli F, Caumes E, Chappuis F, Cramer JP et al: Differential diagnosis of illness in travelers arriving from Sierra Leone, Liberia, or Guinea: a cross-sectional study from the GeoSentinel Surveillance Network. Ann Intern Med 2015, 162(11):757-764.

21. Boggild AK, Geduld J, Libman M, Ward BJ, McCarthy A, Hajek J, Ghesquiere W, Vincelette J, Kuhn S, Freedman DO et al: Travel-acquired infections in Canada: CanTravNet 2011-2012. Can Commun Dis Rep 2014, 40(16):313-325.

22. Boggild AK, Geduld J, Libman M, Ward BJ, McCarthy AE, Doyle PW, Ghesquiere W, Vincelette J, Kuhn S, Freedman DO et al: Travel-acquired infections and illnesses in Canadians: surveillance report from CanTravNet surveillance data, 2009-2011. Open Med 2014, 8(1):e20-32.

23. Boggild AK, Geduld J, Libman M, Yansouni CP, McCarthy AE, Hajek J, Ghesquiere W, Vincelette J, Kuhn S, Plourde PJ et al: Illness in Canadian travellers and migrants from Brazil: CanTravNet surveillance data, 2013-2016. Can Commun Dis Rep 2016, 42(8):153-157.

24. Bowman S, Salgado C, DeWaay DJ: Dengue Fever Presenting with Hepatitis. The American Journal of the Medical Sciences 2012, 344(4):335-336.

25. Buonsenso D, Barone G, Onesimo R, Calzedda R, Chiaretti A, Valentini P: The re-emergence of dengue virus in non-endemic countries: a case series. BMC Res Notes 2014, 7:596.

26. Cadot L, Segondy M, Foulongne V: Laboratory surveillance of arboviral infections in a southern France region colonized by Aedes albopictus. Epidemiol Infect 2017, 145(4):710-714.

27. Carbajo A, #237, bal, Rubio A, Viani M, #237, Colombo M, #237: The largest dengue outbreak in Argentina and spatial analyses of dengue cases in relation to a control program in a district with sylvan and urban environments. Asian Pacific Journal of Tropical Medicine 2018, 11(3):227-234.

28. CDC: Dengue Hemorrhagic Fever with Shock in an American Traveler. Morbidity and Mortality Weekly Report 1978, 27(48):476-483.

29. CDC: Dengue type 4 infections in U.S. travelers to the Caribbean. MMWR Morb Mortal Wkly Rep 1981, 30(21):249-250.

30. CDC: Imported dengue type 4--Florida. MMWR Morb Mortal Wkly Rep 1982, 30(50-51):622-623.

31. CDC: Imported dengue fever--United States, 1982. MMWR Morb Mortal Wkly Rep 1983, 32(11):145-146.

32. CDC: Imported dengue fever--United States, 1984. MMWR Morb Mortal Wkly Rep 1985, 34(31):488-489.

33. CDC: Epidemiologic Notes and Reports Dengue Fever in U.S. Military Personnel -- Republic of the Philippines. MMWR Morb Mortal Wkly Rep 1985, 34(32):495-496, 501-492.

34. CDC: Imported and indigenous dengue fever--United States, 1986. MMWR Morb Mortal Wkly Rep 1987, 36(33):551-554.

35. CDC: Imported dengue--United States, 1987. MMWR Morb Mortal Wkly Rep 1989, 38(26):463-465.

36. CDC: Imported dengue--United States, 1988. MMWR Morb Mortal Wkly Rep 1990, 39(8):127-128, 133.

37. CDC: Imported dengue--United States, 1989. MMWR Morb Mortal Wkly Rep 1990, 39(41):741-742.

38. CDC: Imported dengue--United States, 1990. MMWR Morb Mortal Wkly Rep 1991, 40(30):519-520.

39. CDC: From the Centers for Disease Control. Imported dengue--United States, 1991. JAMA 1992, 268(15):2000.

40. CDC: Imported dengue--United States, 1992. MMWR Morb Mortal Wkly Rep 1994, 43(6):97-99.

41. CDC: Imported dengue--United States, 1993-1994. MMWR Morb Mortal Wkly Rep 1995, 44(18):353-356.

42. CDC: Imported dengue--United States, 1995. MMWR Morb Mortal Wkly Rep 1996, 45(45):988-991.

43. CDC: From the Centers for Disease Control and Prevention. Imported dengue--United States, 1996. JAMA 1998, 280(13):1132.

44. CDC: From the Centers for Disease Control and Prevention. Imported dengue--United States, 1997 and 1998. JAMA 2000, 283(15):1953-1954.

45. CDC: Imported dengue--United States, 1999 and 2000. MMWR Morb Mortal Wkly Rep 2002, 51(13):281-283.

46. CDC: Travel-associated dengue infections--United States, 2001-2004. MMWR Morb Mortal Wkly Rep 2005, 54(22):556-558.

47. CDC: Travel-Associated Dengue — United States, 2005. Morbidity and Mortality Weekly Report 2006, 55(25):700-702.

48. CDC: Travel-associated Dengue surveillance - United States, 2006-2008. MMWR Morb Mortal Wkly Rep 2010, 59(23):715-719.

49. CDC: Dengue Fever Among U.S. Travelers Returning from the Dominican Republic --- Minnesota and Iowa, 2008. Morbidity and Mortality Weekly Report 2010, 59(21):654-656.

50. CDC: Dengue virus infections among travelers returning from Haiti--Georgia and Nebraska, October 2010. 2011, 60(27):914-917.

51. Chalupa P, Kolarova M, Sojkova N, Januska J: Diagnosis of Imported Dengue Fever in the Czech Republic. Dengue Bulletin 2003, 27:34-38.

52. Chalupa P, Kubek J, Hejlova A: Dengue fever in the Czech Republic. Bratisl Lek Listy 2001, 102(7):322-325.

53. Chappuis F, Justafre JC, Duchunstang L, Loutan L, Taylor WR: Dengue fever and long thoracic nerve palsy in a traveler returning from Thailand. J Travel Med 2004, 11(2):112-114.

54. Chinikar S, Ghiasi SM, Shah-Hosseini N, Mostafavi E, Moradi M, Khakifirouz S, Rasi Varai FS, Rafigh M, Jalali T, Goya MM et al: Preliminary study of dengue virus infection in Iran. Travel Med Infect Dis 2013, 11(3):166-169.

55. Chinikar S, Mojtaba Ghiasi S, Moradi A, Reza Madihi S: Laboratory Detection Facility of Dengue Fever (DF) in Iran: The First Imported Case. the internet journal of infection diseases 2010, 8(1):1-2.

56. Choe YJ, Choe SA, Cho SI: Importation of travel-related infectious diseases is increasing in South Korea: An analysis of salmonellosis, shigellosis, malaria, and dengue surveillance data. Travel Med Infect Dis 2017, 19:22-27.

57. Chong CH, McCaskill ME, Britton PN: Pediatric travelers presenting to an Australian emergency department (2014-2015): A retrospective, cross-sectional analysis. Travel Med Infect Dis 2019, 31:101345.

58. Chuang V, Wong TY, Leung YH, Ma E, Law YL, Tsang O, Chan KM, Tsang I, Que TL, Yung R et al: Review of dengue fever cases in Hong Kong during 1998 to 2005. Hong Kong Med J 2008, 14(3):170-177.

59. Colavita F, Vairo F, Carletti F, Boccardo C, Ferraro F, Iaiani G, Al Moghazi S, Galardo G, Lalle E, Selvaggi C et al: Full-length genome sequence of a dengue serotype 1 virus isolate from a traveler returning from Democratic Republic of Congo to Italy, July 2019. Int J Infect Dis 2020, 92:46-48.

60. Courtney M, Shetty AK: Imported Dengue Fever: an important reemerging disease. Pediatr Emerg Care 2009, 25(11):769-772.

61. Cunha BA, Munoz-Gomez S: Dengue fever in a returning traveller from El Salvador: another cause of a false positive Monospot test. Travel Med Infect Dis 2014, 12(3):293-295.

62. Cunha BA, Raza M: During influenza season: all influenza-like illnesses are not due to influenza: dengue mimicking influenza. J Emerg Med 2015, 48(5):e117-120.

63. Cvjetković D, Mikić S, Hrnjaković-Cvjetković I: Facing dengue fever - our first experience. Vojnosanitetski Pregled 2017, 74(6):590-593.

64. de Laval F, Dia A, Plumet S, Decam C, Leparc Goffart I, Deparis X: Dengue surveillance in the French armed forces: a dengue sentinel surveillance system in countries without efficient local epidemiological surveillance. J Travel Med 2013, 20(4):259-261.

65. Dinu S, Panculescu-Gatej IR, Florescu SA, Popescu CP, Sirbu A, Oprisan G, Badescu D, Franco L, Ceianu CS: Molecular epidemiology of dengue fever cases imported into Romania between 2008 and 2013. Travel Med Infect Dis 2015, 13(1):69-73.

66. Doherty JF, Grant AD, Bryceson AD: Fever as the presenting complaint of travellers returning from the tropics. Qjm 1995, 88(4):277-281.

67. Doi ML, Tatsuno SY, Singh G, Tatsuno EM, Mau MM: Neurological Complications in a Polynesian Traveler with Dengue. Hawaii J Med Public Health 2017, 76(10):275-278.

68. Doolub G: The Returning Traveller: Dengue Fever Report. Case Rep Gastroenterol 2010, 4(3):335-339.

69. Douvoyiannis M, Litman N, Goldman DL: A traveler with rash and thrombocytopenia. Clin Pediatr (Phila) 2009, 48(5):568-571.

70. Duber HC, Kelly SM: Febrile illness in a young traveler: dengue fever and its complications. J Emerg Med 2013, 45(4):526-529.

71. Dumoulin A, Marti H, Panning M, Hatz C, Hirsch HH: Pan-dengue virus detection by PCR for travelers returning from the tropics. J Clin Microbiol 2008, 46(9):3104-3106.

72. Eckerle I, Briciu VT, Ergonul O, Lupse M, Papa A, Radulescu A, Tsiodras S, Tsitou C, Drosten C, Nussenblatt VR et al: Emerging souvenirs-clinical presentation of the returning traveller with imported arbovirus infections in Europe. Clin Microbiol Infect 2018, 24(3):240-245.

73. Eisenhut M, Schwarz TF, Hegenscheid B: Seroprevalence of dengue, chikungunya and Sindbis virus infections in German aid workers. Infection 1999, 27(2):82-85.

74. El-Badry A, El-Beshbishy H, Al Ali K, Al-Hejin A, El-Sayed W: Molecular and seroprevalence of imported dengue virus infection in Al-Madinah, Saudi Arabia. Comparative Clinical Pathology 2013, 22.

75. Erra EO, Korhonen EM, Voutilainen L, Huhtamo E, Vapalahti O, Kantele A: Dengue in travelers: kinetics of viremia and NS1 antigenemia and their associations with clinical parameters. PloS one 2013, 8(6):e65900-e65900.

76. Esposito DH, Han PV, Kozarsky PE, Walker PF, Gkrania-Klotsas E, Barnett ED, Libman M, McCarthy AE, Field V, Connor BA et al: Characteristics and spectrum of disease among ill returned travelers from pre- and post-earthquake Haiti: The GeoSentinel experience. Am J Trop Med Hyg 2012, 86(1):23-28.

77. Fabrizio C, Lepore L, Chironna M, Angarano G, Saracino A: Dengue fever in travellers and risk of local spreading: case reports from Southern Italy and literature update. New Microbiol 2017, 40(1):11-18.

78. Fortuna C, Remoli ME, Rizzo C, Benedetti E, Fiorentini C, Bella A, Argentini C, Farchi F, Castilletti C, Capobianchi MR et al: Imported arboviral infections in Italy, July 2014-October 2015: a National Reference Laboratory report. BMC Infect Dis 2017, 17(1):216.

79. Fuchs I, Bin H, Schlezinger S, Schwartz E: NS1 antigen testing for the diagnosis of dengue in returned Israeli travelers. Journal of Medical Virology 2014, 86(12):2005-2010.

80. Gautret P, Cramer JP, Field V, Caumes E, Jensenius M, Gkrania-Klotsas E, de Vries PJ, Grobusch MP, Lopez-Velez R, Castelli F et al: Infectious diseases among travellers and migrants in Europe, EuroTravNet 2010. Eurosurveillance 2012, 17(26):20205.

81. Gautret P, Mockenhaupt F, Grobusch MP, Rothe C, von Sonnenburg F, van Genderen PJ, Chappuis F, Asgeirsson H, Caumes E, Bottieau E et al: Arboviral and other illnesses in travellers returning from Brazil, June 2013 to May 2016: implications for the 2016 Olympic and Paralympic Games. Euro Surveill 2016, 21(27).

82. Gilbert JD, Higgins G, Byard RW: Unexpected death due to dengue virus infection in a non-endemic area. Australian Journal of Forensic Sciences 2016, 48(3):323-325.

83. Gobbi F, Capelli G, Angheben A, Giobbia M, Conforto M, Franzetti M, Cattelan AM, Raise E, Rovere P, Mulatti P et al: Human and entomological surveillance of West Nile fever, dengue and chikungunya in Veneto Region, Italy, 2010-2012. BMC Infect Dis 2014, 14:60.

84. Goljan J, Myjak P, Nahorski W, Kubica-Biernat B, Felczak-Korzybska I, Kowalczyk D, Kuna A, Kotlowski A: Dengue antibodies in Polish travellers returning from the tropics. Evaluation of serological tests. Int Marit Health 2010, 61(1):36-40.

85. Griffiths KM, Savini H, Brouqui P, Simon F, Parola P, Gautret P: Surveillance of travel-associated diseases at two referral centres in Marseille, France: a 12-year survey. Journal of Travel Medicine 2018, 25(1).

86. Gupta BP, Adhikari A, Rauniyar R, Kurmi R, Upadhya BP, Jha BK, Pandey B, Das Manandhar K: Dengue virus infection in a French traveller to the hilly region of Nepal in 2015: a case report. J Med Case Rep 2016, 10:65.

87. Hafkin B, Kaplan JE, Reed C, Elliott LB, Fontaine R, Sather GE, Kappus K: Reintroduction of dengue fever into the continental United States. I. Dengue surveillance in Texas, 1980. Am J Trop Med Hyg 1982, 31(6):1222-1228.

88. Hashimoto T, Kutsuna S, Maeki T, Tajima S, Takaya S, Katanami Y, Yamamoto K, Takeshita N, Hayakawa K, Kato Y et al: A Case of Dengue Fever Imported from Burkina Faso to Japan in October 2016. Jpn J Infect Dis 2017, 70(6):675-677.

89. Hebbal P, Darwich Y, Fong J, Hagmann SHF, Purswani MU: Nephrotic-range proteinuria in an eight-year-old traveler with severe dengue: Case report and review of the literature. Travel Med Infect Dis 2016, 14(1):45-48.

90. Helbok R, Dent W, Gattringer K, Innerebner M, Schmutzhard E: Imported Dengue fever presenting with febrile diarrhoea: report of two cases. Wien Klin Wochenschr 2004, 116 Suppl 4:58-60.

91. Herbinger KH, Alberer M, Berens-Riha N, Schunk M, Bretzel G, von Sonnenburg F, Nothdurft HD, Loscher T, Beissner M: Spectrum of Imported Infectious Diseases: A Comparative Prevalence Study of 16,817 German Travelers and 977 Immigrants from the Tropics and Subtropics. Am J Trop Med Hyg 2016, 94(4):757-766.

92. Hesse EM, Martinez LJ, Jarman RG, Lyons AG, Eckels KH, De La Barrera RA, Thomas SJ: Dengue Virus Exposures Among Deployed U.S. Military Personnel. Am J Trop Med Hyg 2017, 96(5):1222-1226.

93. Hochedez P, Canestri A, Guihot A, Brichler S, Bricaire F, Caumes E: Management of travelers with fever and exanthema, notably dengue and chikungunya infections. Am J Trop Med Hyg 2008, 78(5):710-713.

94. Hoffmeister B, Suttorp N, Zoller T: The revised dengue fever classification in German travelers: clinical manifestations and indicators for severe disease. Infection 2015, 43(1):21-28.

95. Iannetta M, Lalle E, Musso M, Carletti F, Scorzolini L, D'Abramo A, Chinello P, Castilletti C, Ippolito G, Capobianchi MR et al: Persistent detection of dengue virus RNA in vaginal secretion of a woman returning from Sri Lanka to Italy, April 2017. Euro Surveill 2017, 22(34).

96. Iovine NM, Lednicky J, Cherabuddi K, Crooke H, White SK, Loeb JC, Cella E, Ciccozzi M, Salemi M, Morris JG, Jr.: Coinfection With Zika and Dengue-2 Viruses in a Traveler Returning From Haiti, 2016: Clinical Presentation and Genetic Analysis. Clin Infect Dis 2017, 64(1):72-75.

97. Ito M, Yamada K, Takasaki T, Pandey B, Nerome R, Tajima S, Morita K, Kurane I: Phylogenetic analysis of dengue viruses isolated from imported dengue patients: possible aid for determining the countries where infections occurred. J Travel Med 2007, 14(4):233-244.

98. Iwamoto I, Muta N, Nakajima Y, Murakami F, Totogawa S, et al. : A case of imported dengue fever. . Trop Med 1973, 15:225-230.

99. J Cotter C, Tufa A, Johnson S, Matai'a M, Sciulli R, R Ryff K, Thane Hancock W, Whelen C, Sharp T, Scott Anesi M: Outbreak of Dengue Virus Type 2 - American Samoa, November 2016-October 2018. MMWR Morbidity and mortality weekly report 2018, 67:1319-1322.

100. Jacobs MG, Brook MG, Weir WR, Bannister BA: Dengue haemorrhagic fever: a risk of returning home. BMJ 1991, 302(6780):828-829.

101. Jelinek T, Dobler G, Hölscher M, Löscher T, Nothdurft H-D: Prevalence of Infection With Dengue Virus Among International Travelers. JAMA Internal Medicine 1997, 157(20):2367-2370.

102. Jelinek T, Muhlberger N, Harms G, Corachan M, Grobusch MP, Knobloch J, Bronner U, Laferl H, Kapaun A, Bisoffi Z et al: Epidemiology and clinical features of imported dengue fever in Europe: sentinel surveillance data from TropNetEurop. Clin Infect Dis 2002, 35(9):1047-1052.

103. Jensenius M, Berild D, Ormaasen V, Maehlen J, Lindegren G, Falk KI: Fatal subarachnoidal haemorrhage in a Norwegian traveller with dengue virus infection. Scand J Infect Dis 2007, 39(3):272-274.

104. Jensenius M, Han PV, Schlagenhauf P, Schwartz E, Parola P, Castelli F, von Sonnenburg F, Loutan L, Leder K, Freedman DO et al: Acute and potentially life-threatening tropical diseases in western travelers--a GeoSentinel multicenter study, 1996-2011. Am J Trop Med Hyg 2013, 88(2):397-404.

105. Jerome H, Taylor C, Sreenu VB, Klymenko T, Filipe ADS, Jackson C, Davis C, Ashraf S, Wilson-Davies E, Jesudason N et al: Metagenomic next-generation sequencing aids the diagnosis of viral infections in febrile returning travellers. J Infect 2019, 79(4):383-388.

106. Jiang L, Wu X, Wu Y, Bai Z, Jing Q, Luo L, Dong Z, Yang Z, Xu Y, Cao Y et al: Molecular epidemiological and virological study of dengue virus infections in Guangzhou, China, during 2001-2010. Virol J 2013, 10:4.

107. Jones JM, Lopez B, Adams L, Galvez FJ, Nunez AS, Santillan NA, Plante L, Hemme RR, Casal M, Hunsperger EA et al: Binational Dengue Outbreak Along the United States-Mexico Border - Yuma County, Arizona, and Sonora, Mexico, 2014. MMWR Morb Mortal Wkly Rep 2016, 65(19):495-499.

108. Korhonen EM, Huhtamo E, Virtala A-MK, Kantele A, Vapalahti O: Approach to non-invasive sampling in dengue diagnostics: Exploring virus and NS1 antigen detection in saliva and urine of travelers with dengue. Journal of Clinical Virology 2014, 61(3):353-358.

109. Kuan MM, Chang FY: Airport sentinel surveillance and entry quarantine for dengue infections following a fever screening program in Taiwan. BMC Infect Dis 2012, 12:182.

110. Kuan MM, Lin T, Chuang JH, Wu HS: Epidemiological trends and the effect of airport fever screening on prevention of domestic dengue fever outbreaks in Taiwan, 1998-2007. Int J Infect Dis 2010, 14(8):e693-697.

111. Kumar S, Iuga A, Jean R: Cardiac tamponade in a patient with dengue fever and lupus nephritis: a case report. J Intensive Care Med 2010, 25(3):175-178.

112. Kuna A, Bykowska M, Kulawiak N, Biernat B, Szostakowska B, Nahorski WL, Pawlowski W, Chomicz L: Clinico-laboratory profile of dengue patients returning from tropical areas to Poland during 2010-15. J Vector Borne Dis 2016, 53(3):234-239.

113. Kuna A, Wroczynska A, Gajewski M, Felczak-Korzybska I, Nahorski WL: A case of acalculous cholecystitis in the course of dengue fever in a traveller returned from Brazil. Int Marit Health 2016, 67(1):38-41.

114. Kurane I, Takasaki T, Yamada K: Trends in flavivirus infections in Japan. Emerg Infect Dis 2000, 6(6):569-571.

115. Kusama Y, Ito K, Tajima S, Kutsuna S: A pediatric case of imported dengue hemorrhagic fever in Japan. J Gen Fam Med 2017, 18(6):414-417.

116. Laferl H, Szell M, Bischof E, Wenisch C: Imported dengue fever in Austria 1990-2005. Travel Med Infect Dis 2006, 4(6):319-323.

117. Langgartner J, Audebert F, Schölmerich J, Glück T: Dengue Virus Infection Transmitted by Needle Stick Injury. Journal of Infection 2002, 44(4):269-270.

118. Lau CL, Weinstein P, Slaney D: Dengue surveillance by proxy: travellers as sentinels for outbreaks in the Pacific Islands. Epidemiol Infect 2013, 141(11):2328-2334.

119. Lawn SD, Tilley R, Lloyd G, Finlayson C, Tolley H, Newman P, Rice P, Harrison TS: Dengue hemorrhagic fever with fulminant hepatic failure in an immigrant returning to Bangladesh. Clin Infect Dis 2003, 37(1):e1-4.

120. Leder K, Mutsch M, Schlagenhauf P, Luxemburger C, Torresi J: Seroepidemiology of dengue in travellers: a paired sera analysis. Travel Med Infect Dis 2013, 11(4):210-213.

121. Liu W, Pickering P, Duchene S, Holmes EC, Aaskov JG: Highly Divergent Dengue Virus Type 2 in Traveler Returning from Borneo to Australia. Emerg Infect Dis 2016, 22(12):2146-2148.

122. Loconsole D, Metallo A, De Robertis AL, Morea A, Quarto M, Chironna M: Seroprevalence of Dengue Virus, West Nile Virus, Chikungunya Virus, and Zika Virus in International Travelers Attending a Travel and Migration Center in 2015-2017, Southern Italy. Vector Borne Zoonotic Dis 2018, 18(6):331-334.

123. Lopez-Velez R, Perez-Casas C, Vorndam AV, Rigau J: Dengue in Spanish travelers returning from the tropics. Eur J Clin Microbiol Infect Dis 1996, 15(10):823-826.

124. Lustig Y, Wolf D, Halutz O, Schwartz E: An outbreak of dengue virus (DENV) type 2 Cosmopolitan genotype in Israeli travellers returning from the Seychelles, April 2017. Euro Surveill 2017, 22(26).

125. Lyerla R, Rigau-Perez JG, Vorndam AV, Reiter P, George AM, Potter IM, Gubler DJ: A dengue outbreak among camp participants in a Caribbean island, 1995. J Travel Med 2000, 7(2):59-63.

126. Maguire T: Do Ross River and dengue viruses pose a threat to New Zealand? N Z Med J 1994, 107(989):448-450.

127. Malcolm RL, Hanna JN, Phillips DA: The timeliness of notification of clinically suspected cases of dengue imported into north Queensland. Aust N Z J Public Health 1999, 23(4):414-417.

128. Malison MD, Waterman SH: Dengue fever in the United States. A report of a cluster of imported cases and review of the clinical, epidemiologic, and public health aspects of the disease. JAMA 1983, 249(4):496-500.

129. Matsui T, Kinoshita N, Maeki T, Kutsuna S, Nakamura K, Nakamoto T, Ishikane M, Tajima S, Kato F, Taniguchi S et al: Dengue Virus Type 2 Infection in a Traveler Returning from Saudi Arabia to Japan. Jpn J Infect Dis 2019, 72(5):340-342.

130. McCarthy MA, Carpenter D, Goyette M, Nguyen DT: Dengue fever in Canada. Can Commun Dis Rep 1995, 21(20):185-187.

131. Mendonca MCL, Mares-Guia MA, Rodrigues C, Santos CCD, Chalhoub FLL, Araujo ESM, Chieppe AO, Nogueira RMR, Filippis AMB: Imported case of Dengue virus 3 genotype I in Rio de Janeiro state, Brazil. Mem Inst Oswaldo Cruz 2018, 113(8):e180036.

132. Mills GD, Jones PD: Clinical spectrum of dengue fever in travellers. N Z Med J 1991, 104(913):228-230.

133. Moncayo AC, Baumblatt J, Thomas D, Harvey KA, Atrubin D, Stanek D, Sotir M, Hunsperger E, Munoz-Jordan JL, Jentes ES et al: Dengue among American missionaries returning from Jamaica, 2012. Am J Trop Med Hyg 2015, 92(1):69-71.

134. Mustaf AS, Elbishbishi EA, Grover S, Pacsa AS, Al-Enezi AA, Chaturvedi UC: A study of dengue imported to Kuwait during 1997-1999. Acta Virol 2001, 45(2):125-128.

135. Mutoh Y, Moriya A, Yasui Y, Saito N, Takasaki T, Hiramatsu S, Izuchi T, Umemura T, Ichihara T: Two Cases of Dengue Virus Type 2 (DENV-2) Infection in a Japanese Couple Returning from the Maldives during the 2018 Dengue Outbreak. Jpn J Infect Dis 2020, 73(1):58-60.

136. Napoli C, Salcuni P, Pompa MG, Declich S, Rizzo C: Estimated imported infections of Chikungunya and Dengue in Italy, 2008 to 2011. J Travel Med 2012, 19(5):294-297.

137. Neumayr A, Munoz J, Schunk M, Bottieau E, Cramer J, Calleri G, Lopez-Velez R, Angheben A, Zoller T, Visser L et al: Sentinel surveillance of imported dengue via travellers to Europe 2012 to 2014: TropNet data from the DengueTools Research Initiative. Euro Surveill 2017, 22(1).

138. O'Neill SL, Ryan PA, Turley AP, Wilson G, Retzki K, Iturbe-Ormaetxe I, Dong Y, Kenny N, Paton CJ, Ritchie SA et al: Scaled deployment of Wolbachia to protect the community from dengue and otherآ  Aedes transmitted arboviruses. Gates Open Res 2018, 2:36.

139. Okada K, Morita R, Egawa K, Hirai Y, Kaida A, Shirano M, Kubo H, Goto T, Yamamoto SP: Dengue Virus Type 1 Infection in Traveler Returning from Tanzania to Japan, 2019. Emerg Infect Dis 2019, 25(9):1782-1784.

140. Olivero RM, Hamer DH, MacLeod WB, Benoit CM, Sanchez-Vegas C, Jentes ES, Chen LH, Wilson ME, Marano N, Yanni EA et al: Dengue Virus Seroconversion in Travelers to Dengue-Endemic Areas. Am J Trop Med Hyg 2016, 95(5):1130-1136.

141. Parola P, Soula G, Gazin P, Foucault C, Delmont J, Brouqui P: Fever in travelers returning from tropical areas: prospective observational study of 613 cases hospitalised in Marseilles, France, 1999-2003. Travel Med Infect Dis 2006, 4(2):61-70.

142. Parreira R, Centeno-Lima S, Lopes A, Portugal-Calisto D, Constantino A, Nina J: Dengue virus serotype 4 and chikungunya virus coinfection in a traveller returning from Luanda, Angola, January 2014. Euro Surveill 2014, 19(10).

143. Parreira R, Conceicao C, Centeno-Lima S, Marques N, Saraiva da Cunha J, Abreu C, Sa L, Sarmento A, Atouguia J, Moneti V et al: Angola's 2013 dengue outbreak: clinical, laboratory and molecular analyses of cases from four Portuguese institutions. J Infect Dev Ctries 2014, 8(9):1210-1215.

144. Patey O, Ollivaud L, Breuil J, Lafaix C: Unusual neurologic manifestations occurring during dengue fever infection. Am J Trop Med Hyg 1993, 48(6):793-802.

145. Paulo CO, Ze-Ze L, Jordao S, Pena ER, Neves I, Alves MJ: Dengue virus serotype 3 and Chikungunya virus co-infection in a traveller returning from India to Portugal, November 2016. IDCases 2017, 9:30-33.

146. Pincus LB, Grossman ME, Fox LP: The exanthem of dengue fever: Clinical features of two US tourists traveling abroad. J Am Acad Dermatol 2008, 58(2):308-316.

147. Poudel A, Shah Y, Khatri B, Joshi D, R Bhatta D, Pandey B: The burden of dengue infection in some vulnerable regions of Nepal. Nepal Med Coll J 2012, 14:114-117.

148. Quinn EJ, Cheong AH, Calvert JK, Higgins G, Hahesy T, Gordon DL, Carr JM: Clinical Features and Laboratory Findings of Travelers Returning to South Australia with Dengue Virus Infection. Trop Med Infect Dis 2018, 3(1).

149. Rawlings JA, Hendricks KA, Burgess CR, Campman RM, Clark GG, Tabony LJ, Patterson MA: Dengue surveillance in Texas, 1995. Am J Trop Med Hyg 1998, 59(1):95-99.

150. Redondo-Bravo L, Ruiz-Huerta C, Gomez-Barroso D, Sierra-Moros MJ, Benito A, Herrador Z: Imported dengue in Spain: a nationwide analysis with predictive time series analyses. J Travel Med 2019, 26(8).

151. Ribeiro E, Kassab S, Pistone T, Receveur MC, Fialon P, Malvy D: Primary dengue fever associated with hemophagocytic syndrome: a report of three imported cases, Bordeaux, France. Intern Med 2014, 53(8):899-902.

152. Riddell A, Babiker ZO: Imported dengue fever in East London: a 6-year retrospective observational study. J Travel Med 2017, 24(3).

153. Rocco IM, Kavakama BB, Santos CL: First isolation of dengue 3 in Brazil from an imported case. Rev Inst Med Trop Sao Paulo 2001, 43(1):55-57.

154. Rocklov J, Lohr W, Hjertqvist M, Wilder-Smith A: Attack rates of dengue fever in Swedish travellers. Scand J Infect Dis 2014, 46(6):412-417.

155. Sahni LC, Fischer RSB, Gorchakov R, Berry RM, Payne DC, Murray KO, Boom JA: Arboviral Surveillance among Pediatric Patients with Acute Febrile Illness in Houston, Texas. Am J Trop Med Hyg 2018, 99(2):413-416.

156. Sang S, Chen B, Wu H, Yang Z, Di B, Wang L, Tao X, Liu X, Liu Q: Dengue is still an imported disease in China: a case study in Guangzhou. Infect Genet Evol 2015, 32:178-190.

157. Schmidt-Chanasit J, Emmerich P, Tappe D, Günther S, Schmidt S, Wolff D, Hentschel K, Sagebiel D, Schöneberg I, Stark K et al: Autochthonous dengue virus infection in Japan imported into Germany, September 2013. Eurosurveillance 2014, 19(3):20681.

158. Schultze D, Berendonk C, Ammann T, Niedrig M: Isolation of dengue virus serotype 1 from the blood of a Swiss traveler prior to seroconversion. Infection 2002, 30(4):237-239.

159. Schwartz E: Study of Dengue Fever among Israeli Travellers to Thailand. Dengue Bulletin 2002, 26:162-167.

160. Schwartz E, Meltzer E, Mendelson M, Tooke A, Steiner F, Gautret P, Friedrich-Jaenicke B, Libman M, Bin H, Wilder-Smith A et al: Detection on four continents of dengue fever cases related to an ongoing outbreak in Luanda, Angola, March to May 2013. Euro Surveill 2013, 18(21).

161. Schwartz E, Weld LH, Wilder-Smith A, von Sonnenburg F, Keystone JS, Kain KC, Torresi J, Freedman DO, GeoSentinel Surveillance N: Seasonality, annual trends, and characteristics of dengue among ill returned travelers, 1997-2006. Emerg Infect Dis 2008, 14(7):1081-1088.

162. Schwarz TF, Jager G, Gilch S: Imported dengue virus infections in German tourists. Zentralbl Bakteriol 1995, 282(4):533-536.

163. Schwarz TF, Jager G, Gilch S, Pauli C, Eisenhut M, Nitschko H, Hegenscheid B: Travel-related vector-borne virus infections in Germany. Arch Virol Suppl 1996, 11:57-65.

164. Sessions OM, Khan K, Hou Y, Meltzer E, Quam M, Schwartz E, Gubler DJ, Wilder-Smith A: Exploring the origin and potential for spread of the 2013 dengue outbreak in Luanda, Angola. Glob Health Action 2013, 6:21822.

165. Shahhosseini N, Chinikar S, Nowotny N, Fooks AR, Schmidt-Chanasit J: Genetic analysis of imported dengue virus strains by Iranian travelers. Asian Pacific Journal of Tropical Disease 2016, 6(11):850-853.

166. Sharp TW, DeFraites RF, Thornton SA, Burans JP, Wallace MR: Illness in Journalists and Relief Workers Involved in International Humanitarian Assistance Efforts in Somalia, 1992-93. J Travel Med 1995, 2(2):70-76.

167. Sharp TW, Wallace MR, Hayes CG, Sanchez JL, DeFraites RF, Arthur RR, Thornton SA, Batchelor RA, Rozmajzl PJ, Hanson RK et al: Dengue fever in U.S. troops during Operation Restore Hope, Somalia, 1992-1993. Am J Trop Med Hyg 1995, 53(1):89-94.

168. Shirtcliffe P, Cameron E, Nicholson KG, Wiselka MJ: Don't forget dengue! Clinical features of dengue fever in returning travellers. J R Coll Physicians Lond 1998, 32(3):235-237.

169. Shourick J, Dinh A, Matt M, Salomon J, Davido B: Severe neutropenia revealing a rare presentation of dengue fever: a case report. BMC Res Notes 2017, 10(1):415.

170. Sohail A, McGuinness SL, Lightowler R, Leder K, Jomon B, Bain CA, Peleg AY: Spectrum of illness among returned Australian travellers from Bali, Indonesia: a 5-year retrospective observational study. Intern Med J 2019, 49(1):34-40.

171. Soni H, Gandhi V, Varma S, Kaur D, Epelbaum O: A 47-year-old returning traveler with shock. Chest 2015, 147(1):e8-e12.

172. Stephan C, Allwinn R, Brodt HR, Knupp B, Preiser W, Just-Nubling G: Travel-acquired dengue infection: clinical spectrum and diagnostic aspects. Infection 2002, 30(4):225-228.

173. Stephenson I, Roper J, Fraser M, Nicholson K, Wiselka M: Dengue fever in febrile returning travellers to a UK regional infectious diseases unit. Travel Med Infect Dis 2003, 1(2):89-93.

174. Stienlauf S, Segal G, Sidi Y, Schwartz E: Epidemiology of Travel-Related Hospitalization. Journal of Travel Medicine 2006, 12(3):136-141.

175. Suzuki T, Kutsuna S, Taniguchi S, Tajima S, Maeki T, Kato F, Lim CK, Saijo M, Tsuboi M, Yamamoto K et al: Dengue Virus Exported from Cote d'Ivoire to Japan, June 2017. Emerg Infect Dis 2017, 23(10).

176. Tai A, Robosa R, Padiglione AA, Dalpatadu C, Korman TM: Dengue fever in travellers: are we missing warning signs of severe dengue in a non-endemic setting? Med J Aust 2016, 204(7):267.

177. Tai AY, McGuinness SL, Robosa R, Turner D, Huang GK, Leder K, Korman TM, Thevarajan I, Stewardson AJ, Padiglione AA et al: Management of dengue in Australian travellers: a retrospective multicentre analysis. Med J Aust 2017, 206(7):295-300.

178. Takasaki T: Imported dengue fever/dengue hemorrhagic fever cases in Japan. Trop Med Health 2011, 39(4 Suppl):13-15.

179. Tavakolipoor P, Schmidt-Chanasit J, Burchard GD, Jordan S: Clinical features and laboratory findings of dengue fever in German travellers: A single-centre, retrospective analysis. Travel Med Infect Dis 2016, 14(1):39-44.

180. Teichmann D, Gobels K, Niedrig M, Grobusch MP: Dengue virus infection in travellers returning to Berlin, Germany: clinical, laboratory, and diagnostic aspects. Acta Trop 2004, 90(1):87-95.

181. Terzian C, Sheek-Hussein M, Nagelkerke N: The first confirmed case of imported dengue fever in the United Arab Emirates: Clinical, public health, and epidemiological aspects. Emirates Medical Journal 2007, 25(1):61-64.

182. Thai KTD, Wismeijera JA, van Vugta M, Wolthers KC, J de Vriesa P: Dengue fever among ill-returned travellers and concurrent infection by two dengue virus serotypes. Dengue Bulletin 2009, 33:60-69.

183. Thomas DL, Santiago GA, Abeyta R, Hinojosa S, Torres-Velasquez B, Adam JK, Evert N, Caraballo E, Hunsperger E, Munoz-Jordan JL et al: Reemergence of Dengue in Southern Texas, 2013. Emerg Infect Dis 2016, 22(6):1002-1007.

184. Trofa AF, DeFraites RF, Smoak BL, Kanesa-thasan N, King AD, Burrous JM, MacArthy PO, Rossi C, Hoke CH, Jr.: Dengue fever in US military personnel in Haiti. JAMA 1997, 277(19):1546-1548.

185. Trojanek M, Maixner J, Sojkova N, Kyncl J, Rohacova H, Maresova V, Stejskal F: Dengue fever in Czech travellers: A 10-year retrospective study in a tertiary care centre. Travel Med Infect Dis 2016, 14(1):32-38.

186. Tsuboi M, Kutsuna S, Maeki T, Taniguchi S, Tajima S, Kato F, Lim CK, Saijo M, Takaya S, Katanami Y et al: Dengue Virus Type 2 in Travelers Returning to Japan from Sri Lanka, 2017. Emerg Infect Dis 2017, 23(11).

187. Tumioto GL, Gregianini TS, Dambros BP, Cestari BC, Alves Nunes ZM, Veiga AB: Laboratory surveillance of dengue in Rio Grande do Sul, Brazil, from 2007 to 2013. PLoS One 2014, 9(8):e104394.

188. Vainio K, Noraas S, Holmberg M, Fremstad H, Wahlstrom M, Anestad G, Dudman S: Fatal and mild primary dengue virus infections imported to Norway from Africa and south-east Asia, 2008-2010. Euro Surveill 2010, 15(38).

189. Vasquez V, Haddad E, Perignon A, Jaureguiberry S, Brichler S, Leparc-Goffart I, Caumes E: Dengue, chikungunya, and Zika virus infections imported to Paris between 2009 and 2016: Characteristics and correlation with outbreaks in the French overseas territories of Guadeloupe and Martinique. Int J Infect Dis 2018, 72:34-39.

190. Vene S, Mangiafico J, Niklasson B: Indirect immunofluorescence for serological diagnosis of dengue virus infections in Swedish patients. Clin Diagn Virol 1995, 4(1):43-50.

191. Verschueren J, Cnops L, van Esbroeck M: Twelve years of dengue surveillance in Belgian travellers and significant increases in the number of cases in 2010 and 2013. Clin Microbiol Infect 2015, 21(9):867-872.

192. Viennet E, Ritchie SA, Faddy HM, Williams CR, Harley D: Epidemiology of dengue in a high-income country: a case study in Queensland, Australia. Parasites & Vectors 2014, 7(1):379.

193. Vinner L, Domingo C, Ostby AC, Rosenberg K, Fomsgaard A: Cases of travel-acquired dengue fever in Denmark 2001-2009. Clin Microbiol Infect 2012, 18(2):171-176.

194. Visser JT, Edwards CA: Dengue Fever, Tuberculosis, Human Immunodeficiency Virus, and Hepatitis C Virus Conversion in a Group of Long‐Term Development Aid Workers. Journal of Travel Medicine 2013, 20(6):361-367.

195. Visser JT, Narayanan A, Campbell B: Strongyloides, Dengue Fever, and Tuberculosis Conversions in New Zealand Police Deploying Overseas. Journal of Travel Medicine 2012, 19(3):178-182.

196. Wang Y, Wang X, Liu X, Ren R, Zhou L, Li C, Tu W, Ni D, Li Q, Feng Z et al: Epidemiology of Imported Infectious Diseases, China, 2005-2016. Emerg Infect Dis 2018, 25(1):33-41.

197. Warne B, Weld LH, Cramer JP, Field VK, Grobusch MP, Caumes E, Jensenius M, Gautret P, Schlagenhauf P, Castelli F et al: Travel-related infection in European travelers, EuroTravNet 2011. J Travel Med 2014, 21(4):248-254.

198. Wichmann O, Gascon J, Schunk M, Puente S, Siikamaki H, Gjorup I, Lopez-Velez R, Clerinx J, Peyerl-Hoffmann G, Sundoy A et al: Severe dengue virus infection in travelers: risk factors and laboratory indicators. J Infect Dis 2007, 195(8):1089-1096.

199. Wichmann O, Lauschke A, Frank C, Shu PY, Niedrig M, Huang JH, Stark K, Jelinek T: Dengue antibody prevalence in German travelers. Emerg Infect Dis 2005, 11(5):762-765.

200. Wichmann O, Muhlberger N, Jelinek T: Dengue - The Underestimated Risk in Travellers. Dengue Bulletin 2003, 27:126-137.

201. Wittesjo B, Eitrem R, Niklasson B: Dengue fever among Swedish tourists. Scand J Infect Dis 1993, 25(6):699-704.

202. Woodruff AW, Bowen ET, Platt GS: Viral infections in travellers from tropical Africa. Br Med J 1978, 1(6118):956-958.

203. Wu Y, Lien J, Chen HY: Recent Outbreak of Dengue in Taiwan. Tropical Medicine 1994, 35(4):201-207.

204. Yamada KI, Takasaki T, Nawa M, Nakayama M, Arai YT, Yabe S, Kurane I: The features of imported dengue fever cases from 1996 to 1999. Jpn J Infect Dis 1999, 52(6):257-259.

205. Yang CF, Chang SF, Hsu TC, Su CL, Wang TC, Lin SH, Yang SL, Lin CC, Shu PY: Molecular characterization and phylogenetic analysis of dengue viruses imported into Taiwan during 2011-2016. PLoS Negl Trop Dis 2018, 12(9):e0006773.

206. Yuan B, Nishiura H: Estimating the actual importation risk of dengue virus infection among Japanese travelers. PLoS One 2018, 13(6):e0198734.

207. Yue Y, Liu X, Xu M, Ren D, Liu Q: Epidemiological dynamics of dengue fever in mainland China, 2014-2018. Int J Infect Dis 2019, 86:82-93.

208. Zavattoni M, Rovida F, Campanini G, Percivalle E, Sarasini A, Cristini G, Tomasoni LR, Castelli F, Baldanti F: Miscarriage following dengue virus 3 infection in the first six weeks of pregnancy of a dengue virus-naive traveller returning from Bali to Italy, April 2016. Euro Surveill 2016, 21(31).

209. Zea D, Foley K, Carey J: Myocarditis in a traveler returning from the Dominican Republic: an unusual presentation of dengue fever. Am J Trop Med Hyg 2014, 91(1):156-158.

210. Zvereva NN, Saifullin MA, Sayfullin RF, Erovichenkov AA, Bazarova MV, Pshenichnaya NY: Epidemiological and etiological features of travel-related febrile illnesses in hospitalized Russian children and adults: A single-centre, retrospective analysis in Moscow. Travel Med Infect Dis 2020, 34:101447.
